# The cellular and molecular basis of the spur development in *Impatiens uliginosa*

**DOI:** 10.1093/hr/uhae015

**Published:** 2024-01-12

**Authors:** Yang Li, Wu-lue Huang, Xin-yi Li, Ying-duo Zhang, Dan-chen Meng, Chun-mei Wei, Mei-juan Huang, Hai-quan Huang

**Affiliations:** College of Landscape Architecture and Horticulture Sciences, Southwest Research Center for Engineering Technology of Landscape Architecture (State Forestry and Grassland Administration), Yunnan Engineering Research Center for Functional Flower Resources and Industrialization, Research and Development Center of Landscape Plants and Horticulture Flowers, Southwest Forestry University, Kunming, Yunnan, 650224, China; School of Art and Design, Lanzhou Jiaotong University, Lanzhou 730070, China; College of Landscape Architecture and Horticulture Sciences, Southwest Research Center for Engineering Technology of Landscape Architecture (State Forestry and Grassland Administration), Yunnan Engineering Research Center for Functional Flower Resources and Industrialization, Research and Development Center of Landscape Plants and Horticulture Flowers, Southwest Forestry University, Kunming, Yunnan, 650224, China; College of Landscape Architecture and Horticulture Sciences, Southwest Research Center for Engineering Technology of Landscape Architecture (State Forestry and Grassland Administration), Yunnan Engineering Research Center for Functional Flower Resources and Industrialization, Research and Development Center of Landscape Plants and Horticulture Flowers, Southwest Forestry University, Kunming, Yunnan, 650224, China; Department of Biodiversity Conservation, Department of Life Science, Southwest Forestry University, Kunming 650224, China; College of Landscape Architecture and Horticulture Sciences, Southwest Research Center for Engineering Technology of Landscape Architecture (State Forestry and Grassland Administration), Yunnan Engineering Research Center for Functional Flower Resources and Industrialization, Research and Development Center of Landscape Plants and Horticulture Flowers, Southwest Forestry University, Kunming, Yunnan, 650224, China; College of Landscape Architecture and Horticulture Sciences, Southwest Research Center for Engineering Technology of Landscape Architecture (State Forestry and Grassland Administration), Yunnan Engineering Research Center for Functional Flower Resources and Industrialization, Research and Development Center of Landscape Plants and Horticulture Flowers, Southwest Forestry University, Kunming, Yunnan, 650224, China; College of Landscape Architecture and Horticulture Sciences, Southwest Research Center for Engineering Technology of Landscape Architecture (State Forestry and Grassland Administration), Yunnan Engineering Research Center for Functional Flower Resources and Industrialization, Research and Development Center of Landscape Plants and Horticulture Flowers, Southwest Forestry University, Kunming, Yunnan, 650224, China; College of Landscape Architecture and Horticulture Sciences, Southwest Research Center for Engineering Technology of Landscape Architecture (State Forestry and Grassland Administration), Yunnan Engineering Research Center for Functional Flower Resources and Industrialization, Research and Development Center of Landscape Plants and Horticulture Flowers, Southwest Forestry University, Kunming, Yunnan, 650224, China

## Abstract

The nectar spur is an important feature of pollination and ecological adaptation in flowering plants, and it is a key innovation to promote species diversity in certain plant lineages. The development mechanism of spurs varies among different plant taxa. As one of the largest angiosperm genera, we have little understanding of the mechanism of spur development in *Impatiens*. Here, we investigated the initiation and growth process of spurs of *Impatiens uliginosa* based on histology and hormone levels, and the roles of AUXIN BINDING PROTEIN (*ABP*) and extensin (*EXT*) in spur development were explored. Our results indicate that the spur development of *I. uliginosa* is composed of cell division and anisotropic cell elongation. Imbalances in spur proximal-distal cell division lead to the formation of curved structures. Endogenous hormones, such as auxin and cytokinins, were enriched at different developmental stages of spurs. *IuABP* knockdown led to an increase in spur curves and distortion of morphology. *IuEXT* knockdown resulted in reduced spur length and loss of curve and inner epidermal papillae structures. This study provides new insights into the mechanism of spur development in core eudicots.

## Introduction

The floral organs of angiosperms play an important role in reproduction, and their various evolutionary characteristics have clear purposes and adaptive significance; they attract pollinators with their bright colors, fragrant odors, and abundant nectar rewards. The nectar spur, a typical evolutionary floral feature, is a tubular structure extending from petals that have undergone several independent evolutions in angiosperms [1, 2]. Nectar spurs have extensive species diversity in morphology, color, and internal structure [3–5], and play a crucial role in pollination by secreting and storing nectar [6]. In some plant lineages, such as *Aquilegia* and *Linaria*, the evolution of spur length leads to the specialization of pollinators, promoting reproductive isolation and improving pollination efficiency [2, 7–9]. In other plant lineages, such as the *Impatiens*, the pollination system was proven to be generalization rather than specialization [10], and spurs tend to improve pollination efficiency and ecological adaptability through the evolution of curvature [11, 12]. Plant lineages with spurs have an amazing species diversity and faster speciation rate [2, 13]. Therefore, spurs are considered a key innovation [14, 15]. A study on spur development is helpful in understanding the species diversity and evolutionary mechanism of plant lineages.

Spur development consists of cell division and/or cell expansion [16]. In *Aquilegia* and *Centranthus ruber*, this process is divided into two stages. First, cell division at the base of the petal forms the initial spur, after which cell division decreases significantly. The spur attains its final length primarily through anisotropic cell elongation, as the initial spur is only a small fraction of the eventual length [17–19]. The diversity of spur length in *Aquilegia* is primarily influenced by anisotropic cell elongation. However, the study of *Aquilegia rockii* has revealed that differences in spur length within the species are due to variations in cell numbers [20]. In *Pelargonium* and *Linaria*, the difference in spur length cannot be explained by the difference in cell expansion. The differences in spur growth rates caused by changes in cell division and cell number are more important factors, indicating that spur development varies among different species [21, 22].

There is no general molecular mechanism that regulates the formation and development of spurs in angiosperm. Early studies suggest that the *KNOTTED 1*-like homeobox (*KNOX*) gene is expressed in the early stages of spur development in *Linaria* and *Dactylorhiza fuchsii*, regulating spur morphogenesis by promoting cell division and maintaining indeterminate growth [16, 23, 24]. A recent study suggests that the differential expression of *CYCLIN-D3–3* (*CCD33*) and *LONELY GUY 1* (*LOG1*) genes related to cell division and cell cycle on the dorso-ventral side of petals early in spur development may play an important role in the spur formation in *Linaria* [25]. In *Aquilegia*, the C2H2 zinc-finger transcription factor *POPOVICH* (*POP*) is a key gene controlling spur’s presence or absence [26]. *TEOSINTE BRANCHED/CYCLOIDEA/PCF 4* (*TCP4*) sculpts three-dimensional forms of spurs from a two-dimensional primordium by controlling local cell division. In contrast, no *KNOX* gene expression was detected in the transcriptome of early spurs and in in situ hybridization in a broader development stage [27]. Studies on *Tropaeolum* showed that both *TCP* and *KNOX* genes may be involved in spur development and play a crucial role in extreme adaxial-abaxial asymmetry [28]. Furthermore, coregulated gene groups that mediate hormone synthesis or response also participate in spur development. *AUXIN RESPONSE FACTOR ARF6* and *ARF8* regulate spur cell elongation and nectar development in *Aquilegia* [29]. The brassinosteroid pathway gene *BRI1-EMS-SUPRESSOR1/BRASSINAZOLE-RESISTANT1* (*BEH*) regulates the anisotropic elongation and division of spur cells, and the exogenous application of brassinosteroid increased the petal spur length of *Aquilegia* [30].

The genetic mechanism of spur development may vary among different plant lineages. Little is known about spur development in *Impatiens* at present. In previous studies, we divided spur development into three stages based on the length and morphology of the spur in *Impatiens uliginosa*. The early stage lasts about 7 days, with a straight spur reaching 5–8 mm. In the middle stage, the spur develops a curved structure and elongates to 25–28 mm in 4 days. At the anthesis stage, the length and shape of the spur remain stable. We conducted a preliminary exploration of spur development in *Impatiens* through transcriptome sequencing of spurs at these three developmental stages [31]. The results showed that the enrichment function of differentially expressed genes (DEGs) changed with spur development. For instance, in the early stage, ‘regulation of cell cycle’ and ‘auxin-activated signaling pathway’ are some of the most significantly enriched items, while in the middle stage, ‘cell wall organization’ is more prominent. The ‘hormone-mediated signaling pathway’ is the most significant during spur development. These results may reveal the transition between cell division and differentiation, and the key role of hormone regulation.

Candidate genes related to spur development were identified using transcriptome data. Among them, our focus was on the gene AUXIN BINDING PROTEIN (*ABP*), enriched in the auxin-activated signaling pathway, and exhibiting significant differential expression across various tissues and developmental stages. In *Arabidopsis* and tobacco, *ABP* expression is positively correlated with cell size [32–34]. *ABP*-mediated cellular processes (division or expansion) are influenced by auxin concentrations [35]. We hypothesize that *ABP* may regulate the division and elongation of spur cells by mediating the auxin signaling pathway. In addition, the extensin (*EXT*) gene has also attracted our interest, as it has extremely high expression levels in spurs and exhibits significant differences from the limb [31]. Extensins (EXTs) belong to the hydroxyproline-rich glycoprotein (HRGP) superfamily and are important structural proteins in plant cell walls [36, 37]. EXTs affect root hair elongation and lateral root development by regulating the assembly and structure of cell walls in *Arabidopsis*, and this regulation is influenced by auxin induction [38, 39]. Knocking out EXT can cause abnormal cell wall morphology, resulting in germination-defective seedlings with defective root, shoot, and hypocotyl [40, 41].

Here, we studied the spur development process of *I. uliginosa* based on morphology, histocytology, and physiology; the role of *IuABP* and *IuEXT* in spur development was also explored through virus-induced gene silencing (VIGS) technology. The results revealed the cellular and molecular basis of the spur development in *I. uliginosa.*

## Results

### Structural characteristics of spur during morphogenesis

**Figure 1:**
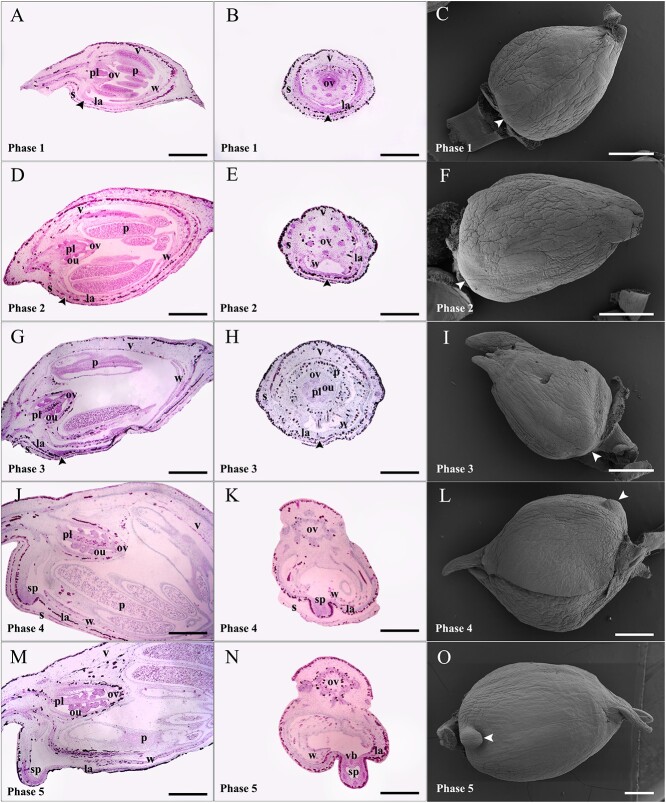
Early spur development of *Impatiens uliginosa*. Paraffin sectioning (longitudinal and transverse sections) was performed on the buds of five consecutive phases to observe the internal structure of spurs, and SEM was used to observe their external morphology. (**A**–**C**) Longitudinal section (A), transverse section (B), and SEM (C) of flower buds in phase 1; spurs and ovules have not developed. (**D**–**F**) Longitudinal section (D), transverse section (E), and SEM (F) of flower buds in phase 2; ovules can be observed, and spurs are still undeveloped. (**G**–**I**) Longitudinal section (G), transverse section (H), and SEM (I) of flower buds in phase 3; spurs are ready to develop, with deep staining (G) and eminence (I) at the growth point. (**J**–**L**) Longitudinal section (J), transverse section (K), and SEM (L) of flower buds in phase 4; the primordial spurs extend from the labellum. (**M**–**O**) Longitudinal section (M), transverse section (N), and SEM (O) of flower buds in phase 5; spurs extend to about 0.4 mm, forming a distinct intracavity and vascular bundles and growing toward the tip of the bud. Arrows indicate spurs or their growth points. la, labellum; ou, ovule; ov, ovary; p, pollen; pl, placenta; s, sepal; sp, spur; v, vexillum; vb, vascular bundle; w, wing. Bars = 500 μm.

To understand spur development and structural features during morphogenesis, we dissected flower buds of *I. uliginosa* across five phases. In phase 1, the sepal, vexillum, wing, labellum, pollen, ovary, and placenta had developed, but ovules were not visible. The spur’s growth point was flat without signs of differentiation (Fig. 1A–C). Phase 2 showed the presence of ovules, yet the spur remained undifferentiated (Fig. 1D–F). During phase 3, a clear deep staining appeared at the spur’s growth point, with a slight protrusion visible under scanning electron microscopy (SEM) (Fig. 1G–I). In phase 4, the labellum thickened, and the spur protruded, forming a shallow cavity (Fig. 1J–L). In phase 5, the spur extended further, forming a distinct cavity, and four vascular bundles were observed in the transverse section. The deep staining on the spur was striking, creating a notable contrast with the limb. It is worth noting that the *I. uliginosa* spur initially grows upward along the ventral midline of the bud, not downward (Fig. 1M–O).

### The spur development of *I. uliginosa* consists of cell division and anisotropic cell elongation

**Figure 2:**
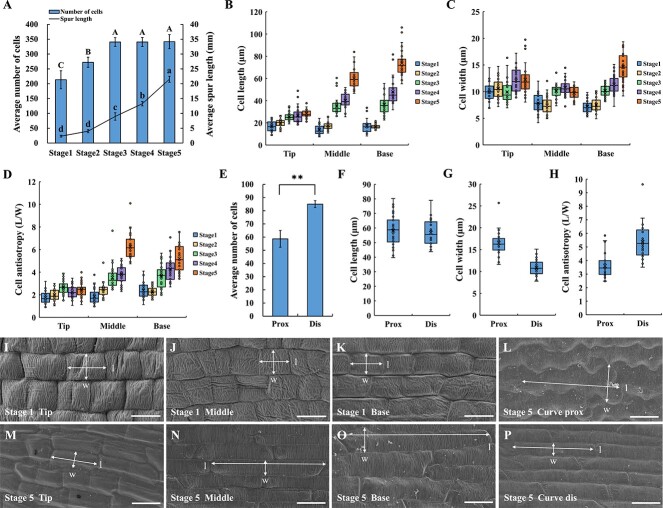
The variation in cell number and cell morphology during spur development. (**A**) Spur length and cell number at five developmental stages, counting cells from the base to the tip of the proximal outer epidermis. (**B**–**D**) Cell length (B), width (C), and anisotropy (D) at five developmental stages, measured at the tip, middle, and base of the spur. (**E**–**H**) Cell number (E), length (F), width (G), and anisotropy (H) on the proximal (prox) and distal (dis) sides of the spur curve in stage 5, with epidermal cells counted and measured within a 5.2 mm range of the curved part. (**I**–**K**) Cell morphology at the tip (I), middle (J), and base (K) of the spur in stage 1. (**L**) Morphology of cells on the proximal (prox) side of the spur curve in stage 5. (**M**–**O**) Cell morphology at the tip (M), middle (N), and base (O) of the spur in stage 5. (**P**) Morphology of cells on the distal (dis) side of the spur curve in stage 5. Error bars represent ± SD in (A) and (E) (*n* = 3). Different letters in (A) indicate statistically significant differences (Tukey’s test: α = 0.05). Asterisks in (E) indicate a significant difference relative to the proximal side (*t* test: **P < 0.01). Data for (B–D) and (F–H) are from three biological replicates, with 10 replicates taken for each biological replicate. Horizontal and vertical lines in (I–P) indicate cell length (l) and width (w), respectively. Bars = 10 μm in (I–K). Bars = 20 μm in (L–P).

To understand the spur development process in *I. uliginosa*, the number, length, and width of epidermal cells were measured at five different developmental stages of spurs. From stage 1 to stage 3, there was a continuous and significant increase in average cell number, rising from 213 to 341, while the average spur length increased by about 7 mm. However, from stage 3 to stage 5, the average number of cells remained almost constant (changing from 341 to 342), while the average spur length increased by about 13 mm. These changes in cell number and spur length suggest that cell division primarily occurs in the early stage, with cell division ceasing after entering the middle stage, making cell number no longer a contributing factor for increased spur length (Fig. 2A; Table S1, see online supplementary material). Epidermal cells of the spur exhibit significant elongation with development (Fig. 2I–K and M–O). The length, width, and anisotropy of cells at the spur tip maintain a gentle growth rate. Although there is little difference in cell length between the middle and base in the first two stages, significant increases occur after stage 2, particularly with the maximum increase in cell length from stage 4 to stage 5. While there is no significant difference in cell length between the three sites during stage 1, as spur development progresses, cells closer to the base exhibit greater elongation (Fig. 2B–D; Table S1, see online supplementary material). Consequently, spur development, once in the middle stage, is primarily driven by the anisotropic cell elongation, with the increase in spur length mainly attributed to cell elongation in the middle and base.

The essence of the spur curve lies in the length difference between the proximal and distal sides. To confirm whether this difference is caused by differences in cell number or length, the number, length, and width of cells on the proximal and distal sides of the spur curve at the anthesis stage were measured. Results showed that within the range of spur curve, the average number of cells in the distal epidermis was significantly greater than that in the proximal epidermis, with a difference of about 26 cells (Fig. 2E). Although the anisotropy of cells on both sides was significantly different due to differences in cell width, their cell length was extremely close (Fig. 2F–H). Therefore, it is speculated that the formation of the spur curve is due to the difference in cell number caused by cell division on the proximal and distal sides. In addition, the cells on both sides showed distinct morphological characteristics, with the proximal cells being wider and exhibiting wavy cell boundaries, while the distal cells being narrower and exhibiting a more regular oblong-like shape (Fig. 2L and P).

### Endogenous hormones change dynamically at different stages of spur development

**Figure 3:**
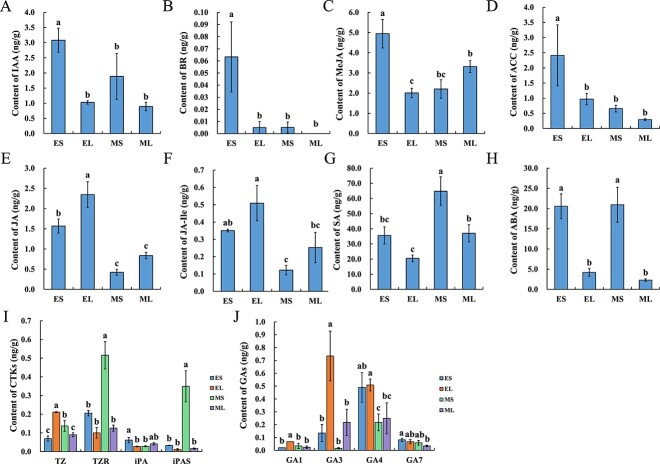
The endogenous hormone content of spurs at different developmental stages and tissues. LC-MS was used to determine the hormone content in the early and middle spur and limb tissues. Each sample contained three biological replicates, and each replicate contained over 100 flower tissues. (**A**) IAA. (**B**) BR. (**C**) MeJA. (**D**) ACC. (**E**) JA. (**F**) JA-Ile. (**G**) SA. (**H**) ABA. (**I**) CTKs. (**J**) GAs. EL, early limb; ES, early spur; ML, middle limb; MS, middle spur. Error bars represent ± SD (N = 3). Different letters indicate statistically significant differences (Tukey’s test: α = 0.05).

The GO and KEGG enrichment analysis of the transcriptome of *I. uliginosa* spur showed that ‘hormone-mediated signaling pathway’ and ‘Plant hormone signal transduction’ pathway were significantly enriched throughout the spur development [31]. To further explore the role of endogenous hormones in spur development, the targeted metabolites of 10 hormones in the early (5 to 8 mm) and middle (13 to 16 mm) spur and limb were detected by liquid chromatography-mass spectrometry (LC–MS) [42]. Hormone levels had significant changes in different tissue and stages. Auxin (IAA), brassinolide (BR), methyl jasmonate (MeJA), and aminocyclopropane carboxylic acid (ACC) were all significantly enriched in the early spur and showed no significant difference in the other three samples (Fig. 3A–D). The content of jasmonic acid (JA) and jasmonic acid-isoleucine (JA-Ile) decreased from the early to middle stages, and the content in spurs was always lower than that in the limb (Fig. 3E and F). Salicylic acid (SA) content significantly increased, and it was higher in the spurs than in the limb (Fig. 3G). The abscisic acid (ABA) content was stable at different stages, but the content in the spur was significantly higher than that in the limb (Fig. 3H).

The content levels of trans-Zeatin riboside (TZR) and isopentenyl adenosine (iPAS) were the highest among the four cytokinins (CTKs); both had the highest content in the middle spur, while there was no significant difference between the other three samples. Trans-zeatin (TZ) increased in spurs, and the early limb had the highest content. Isopentenyl adenine (iPA) and IAA had the same change trend, and these were significantly enriched in the early spurs (Fig. 3I). Both Gibberellin A1 (GA_1_) and GA_3_ had the highest content in the early limb, and there was no significant difference between the other three samples. There was no significant difference in GA4 content between the spur and limb at the same stage, but a significant decline from the early to middle stage was observed. GA_7_ showed no significant differences in different tissues and stages (Fig. 3J).

### Identification of *IuABP* and *IuEXT*

Annotation analysis and NCBI BLAST were utilized to identify the *ABP* and *EXT* homologs from the transcriptome, denoted as *IuABP* and *IuEXT*, respectively [31]. *IuABP* comprises 208 amino acid residues encoded by 573 base pairs, featuring a Cupin domain at positions 64–612 bp. Currently, there is no comprehensive research available on *ABP* gene family members. In maize, five ABP gene family members (*ABP1*, *ABP2*, *ABP3*, *ABP4*, *ABP5*) have been identified, but only *ABP1* and *ABP4* are present in the NCBI database [43, 44]. The Arabidopsis genome contains only one *ABP1* gene [33, 45]. Therefore, to further confirm the orthology of *IuABP*, all available members of the *ABP* gene family were considered to construct a phylogenetic tree with 36 *ABP* genes. These genes were divided into two distinct clades; the first clade included *ABP1*, *ABP4*, *ABP-T85*, and *ABP-T92*, while the second clade contained *ABP19a*, *ABP19b*, and *ABP20*. The *ABP* genes of eight monocotyledonous plants were not independent of all dicotyledonous plants but rather clustered in the first clade, indicating that the *ABP* genes may be conserved in angiosperms. *IuABP* and *Impatiens glandulifera ABP19a* were clustered on a strongly supported branch, suggesting that *IuABP* is an orthology of *ABP19a* (Fig. S1, see online supplementary material).

The total length of *IuEXT* is 1617 bp, encoding 538 amino acids. There are many repeated SPPPPPP motifs in its sequence, with a signal peptide at the N-terminal. Based on these characteristics, it was concluded that *IuEXT* belongs to classical extensin [46, 47]. To further confirm the orthology of *IuEXT*, a phylogenetic tree was constructed for 34 classical *EXT* genes using two monocotyledon species as an outgroup. *IuEXT* and *I. glandulifera EXT2* were clustered on a strongly supported branch, suggesting that *IuEXT* is an orthology of *EXT2* (Fig. S2).

### Silencing of *IuABP* leads to an increase in curved structures and the distortion of spur morphology

**Figure 4:**
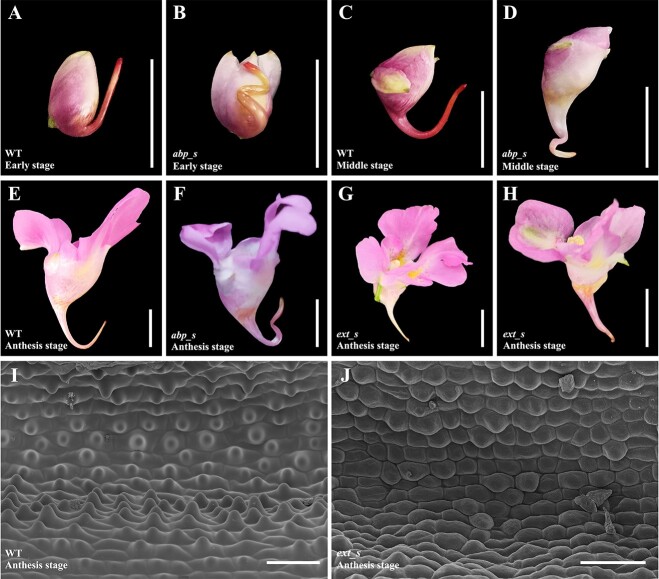
*IuABP* and *IuEXT* silencing led to phenotypic changes in spurs. (**A**) WT flower in the early stage; the curve was not yet formed. (**B**) *abp*_s flower in the early stage with 2–3 twisted curves. (**C**) WT flower in the middle stage, which only had one curve. (**D**) *abp*_s flower in the middle stage. (**E**) WT flower during anthesis. (**F**) *abp*_s flower during anthesis. (**G**–**H**) Strongly *ext*_s flowers during anthesis, with shortened spur and loss of curve, some individuals exhibited a distortion of the entire flower (H). (**I**) Mature WT spurs had distinct papillae on the inner epidermis. (**J**) The inner epidermis of mature *ext*_s spurs; most cells had no papillae. Bars = 10 mm in (A–H). Bars =100 μm in (I–J).

A total of 67 flowers from three plants treated with TRV2-*IuABP* displayed phenotypic changes. Quantitative real-time polymerase chain reaction (qRT-PCR) analysis revealed that the expression level of *IuABP* in TRV2-*IuABP* silenced (*abp*_s) spurs was 13.84% of the wild-type (WT) (Fig. S3A). In WT *I. uliginosa*, the spur forms only one curve in the middle stage, while the *abp*_s spurs exhibited two or three twisted curves in the early stages, causing the entire spur to no longer lie in the same plane. This multicurved morphology was most evident in the early stage, and it persisted until the anthesis stage, although it lessened somewhat with spur development and extension (Fig. 4A–F). We measured the length and curve angle of 25 *abp*_s spurs. The results showed that the length of *abp*_s spurs was not significantly different from that of the WT (Fig. S3C, see online supplementary material), but the curve angles were slightly larger than the WT. As spur development progressed, the angles of the curves tended to increase (Fig. S3D, see online supplementary material).

**Figure 5:**
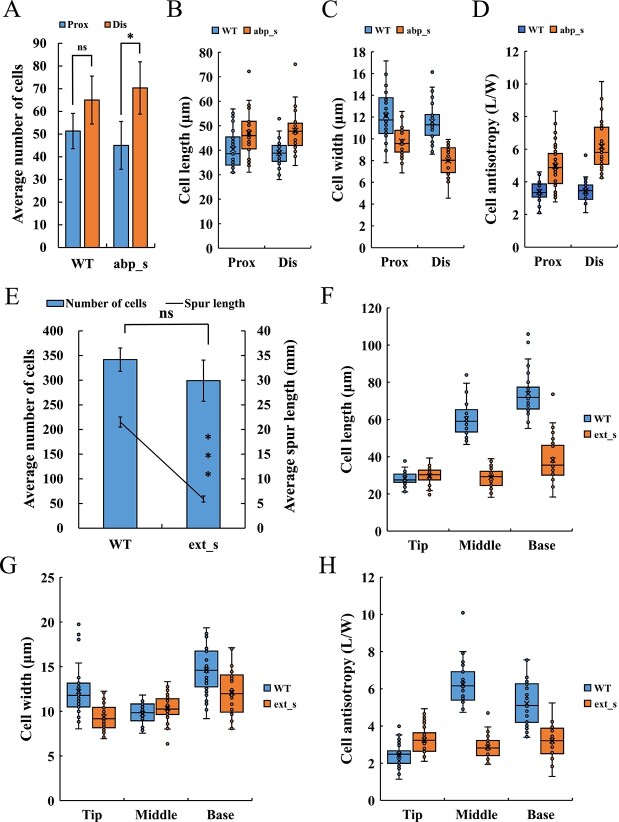
Cell number, length, width, and anisotropy of silenced spurs. (**A**–**D**) Number (A), length (B), width (C), and anisotropy (D) of the proximal (prox) and distal (dis) cells of *abp*_s spurs’ curve compared with those of WT. Epidermal cells within 1.3 mm range of the curved part were counted and measured. (**E**–**H**) Cell number (E), length (F), width (G), and anisotropy (H) of *ext*_s spurs compared with those of WT. Cells were counted along a single line from the base to the tip of the proximal outer epidermis, and the tip, middle, and base of the spur were measured. Error bars in (A) and (E) represent ± SD (*n* = 3). Asterisks in (A) and (E) indicate a significant difference (*t* test: *P < 0.05; ns, not significant). The data for (B–D) and (F–H) came from three biological replicates, with 10 replicates taken for each biological replicate.

A statistical analysis of the number, length, width, and anisotropy of cells on the proximal and distal sides of *abp*_s spurs’ curves was performed. Results showed that the cell number on the proximal side was significantly less than that on the distal side, while there was no significant difference in cell length between these two sides. Proximal cells had less cellular anisotropy due to their wider cell width, but this did not affect the spur length on both sides (Fig. 5A–D;, Fig. S4G and H, see online supplementary material). Therefore, the origin of the curved structure of *abp*_s spurs is consistent with that of WT, which was mainly due to differences in cell number between the two sides. Compared with the WT spurs at the same stage, the cells on both sides of the *abp*_s spurs’ curves have a more significant numerical difference, as well as longer length and smaller width, which leads to significantly higher cell anisotropy than the WT (Fig. 5A–D; Fig. S4E–H, see online supplementary material). In addition, the parenchyma cells at the curved part of *abp*_s spurs showed a local, discontinuous compressed morphology due to irregular distortion (Fig. S4A–D, see online supplementary material).

### *IuEXT* silencing led to a reduction in spur length and disappearance of curve and papillae

Thirty-nine flowers from five plants treated with TRV2-*IuEXT* showed phenotypic changes. The expression level of *IuEXT* in TRV2-*IuEXT* silenced (*ext*_s) spurs was 14.15% of that of WT (Fig. S3B). Notably, *ext*_s spurs displayed a significant reduction in length. We measured the final length of 24 *ext*_s spurs and compared it with that of WT. The average length of *ext*_s spurs was approximately 10.2 mm, which was about 11.8 mm shorter than that of WT (approximately 22 mm) (Fig. S3C, see online supplementary material). In cases of strong silencing, spur lengths were only 5–6 mm. These spurs often lost their curve, straightened, and some individuals exhibited flower shrinkage and distortion (Fig. 4G and H). Furthermore, strongly silenced mature spurs were dissected and compared with WT. In mature WT spurs, the inner epidermal cells displayed prominent papillae, especially those closer to the base (Fig. 4I; Fig. S5A and C, see online supplementary material). However, the inner epidermis of the ext_s spur was relatively smooth and flat, with significantly reduced cell volume compared to WT. The cells were densely arranged, and no prominent papillae were observed. Only a few cells exhibited a slight degree of eminence (Fig. 4J; Fig. S5B and D, see online supplementary material). This suggests that the significant downregulation of *IuEXT* inhibited papillae development and may impact nectar secretion.

To further explore the mechanism of *IuEXT* in regulating spur length, the cell number, length, and width of WT and strongly *ext*_s spurs were measured. Results showed that, although the length of *ext*_s spurs was significantly shorter than that of WT, the cell number was reduced by only about 42 (Fig. 5E). The cell length of the WT spur at the anthesis stage ranged from 28.4 to 73.8 μm (from the tip to the base). Using this standard, the spur length contributed by the reduced cells was in the range of 1.2 to 3.1 mm, which is much smaller than the reduction in length observed in the silenced spur compared to the WT (15.6 mm). Hence, the decrease in cell number is unlikely to be the primary factor in the shortening of spur length. In terms of cell morphology, the cells at the tips of the *ext*_s spurs displayed narrower widths and greater anisotropy compared to the WT, with no significant difference in their length. However, the length and anisotropy of the cells in the middle and base of the *ext*_s spurs significantly decreased (Fig. 5F–H; Fig. S4I–N, see online supplementary material). Therefore, the shortening of the *ext*_s spur was primarily caused by reduced cell elongation in the middle and base of the spur.

## Discussion

### Spur morphogenesis and development in *I. uliginosa* depend on cell division and anisotropic cell elongation

The observation of the anatomical structure of *I. uliginosa* flower buds at various developmental stages revealed deep staining in the spur’s growth point and the primary spur, significantly contrasting with the limb. This indicates a substantial cell accumulation, signifying an active cell division in this region. Just before spur differentiation, a vigorous cell division process commences at the growth point to prepare for spur formation. This localized cell division drives the extension of the spur from the labellum and remains concentrated on the primary spur until it reaches about 9 mm, accounting for 30%–40% of the final length. Cell division on the spur ceases at the 9-mm stage and transitions to cell elongation. At this point, the spur length from the curve to the base (approximately 2.8 mm) is much less than the length from the curve to the tip (about 8.0 mm). By the 22-mm stage (anthesis), the length from the curve to the base (around 10.5 mm) increases significantly compared to the 9 mm stage, while the length from the curve to the tip (about 9.4 mm) shows minimal growth (Fig. S6A and B, see online supplementary material). This variation in spur length aligns with the finding that spur elongation after the middle stage is primarily driven by cell elongation from the middle to the base. It is noteworthy that during the transition from the 4-mm (early stage) to the 9-mm stage (middle stage), both cell division and cell elongation coexist. A key event during this period is the formation of the curve. It is postulated that the gene expression controlling curve formation also initiates the process of cell elongation. Once the curve is formed, cell division comes to a complete halt.

The development process of *I. uliginosa* spur is very similar to that of *Aquilegia* and *C. ruber*, consisting of cell division and anisotropic cell elongation, with cell elongation driving most of the spur growth [17–19]. This process is consistent with previous transcriptome analyses on the functional enrichment of DEGs at different stages [31]. It is worth noting that the spur of *I. uliginosa* experienced two proximal-distal (dorso-ventral) cell division imbalances during development, and this specific spatiotemporal differential cell division constructed the final V shape of the spur. The first time occurs during the early stage of spur differentiation, where the difference in cell number caused by imbalanced division results in a greater dorsal length of the spur, leading to the growth of the primary spur toward the top of the bud (Fig. S6C, see online supplementary material). The second time occurs during the transition from the early stage to the middle stage, where the difference in proximal-distal cell division leads to curve formation, which is similar to that of *Aquilegia brevistyla* [48]. The two sides of the spur curve were subjected to different mechanical stresses, with the proximal side being compressed and the distal side being stretched, resulting in different cell morphology (Fig. 2L and P).

### Hormones affect the spur development

Previous studies provided evidence on the significant enrichment of genes related to the spur development of *I. uliginosa* in hormone-mediated signaling pathway, such as Indol-3-acetic acid (*IuIAA*), *SMALL AUXIN UP RNA* (*IuSAUR*), *IuARF*, and *IuABP*, which showed high expression at the early stage [31]. Influenced by coregulatory gene groups, hormone content changes in different stages and tissues. Auxin usually concentrates on vigorous growth parts and organ formation regions [49, 50], regulating cellular processes, such as cell division, elongation, and differentiation, and affecting the final plant structure [51]. In this study, IAA distribution and concentration showed significant spatial–temporal differences and were associated with cellular processes of spur development. Therefore, it is presumed that auxin and its induced response play a crucial role in spur development. To confirm this hypothesis, exogenous IAA was applied to the outside of the initial spur (approximately 1–2 mm). Results showed that spurs applied with 0.5–10 mM IAA had a faster elongation rate and longer final length (unpublished data). In contrast with *Aquilegia*, IAA application did not cause morphological distortion in *I. uliginosa* due to over and/or uncoordinated lamina tissue proliferation [29], but it only promoted length and growth rate. In addition, hormones, such as GA_4_ and JA_S_, have similar concentration distribution patterns in spurs to auxin, which may form an interactive regulatory network with auxin [52–54]. It is speculated that these hormones not only promote cell division and vigorous early spur growth by maintaining a certain concentration level, but also regulate spur development through mutual response reactions and coregulated downstream genes.

The total CTK content was relatively low in the early stage, and it increased significantly in the middle stage. It seems to be contrary to the process of cell division in the early stage and cell elongation in the middle stage. One possible explanation for this is that the ratio of CTKs to IAA in the spur is relatively low in the early stage and relatively high in the middle stage. Changes in this ratio promote different biological activities, such as inducing callus to form roots when the ratio is low and promoting aboveground formation when the ratio is high [55–57]. It is speculated that during spur development, the growth activities in different stages are regulated by adjusting the ratio of CTKs to IAA, which promotes cell division at a lower ratio in the early stage and cell elongation at a higher ratio in the middle stage. Another possibility is that the CTK types detected were limited and cannot fully reflect the content level in spur. Further validation and supplementary experiments are needed in the future.

Transcriptome analysis showed that DEGs were significantly enriched in the ‘plant-pathogen interaction’ pathway. As the two hormones with the highest levels, SA and ABA may be related to stress resistance during spur development [58, 59]. The increase in SA content in the middle stage may be for flowering preparation [60]. The high level of ABA content in spurs may be related to the enrichment of auxin, and their content maintained a proper balance through interaction and regulation [61, 62]. The ethylene produced by IAA and ABA metabolism led to an increased ACC content, which explains the enrichment of ACC in the early spurs. The GA_1_, GA_3_, and GA_7_ content in the spur was stable and less than that in the limb, while the content of BR in the early spur is extremely low (0.06 ng/g); the content in other tissues approaches zero. It is speculated that these hormones have very limited effects on spur development, contrasting the effect that BR promotes spur development in *Aquilegia* [27, 30].

### *IuABP* and *IuEXT* are involved in the regulation of spur morphology and development by affecting cell division and anisotropic elongation

*IuABP* is an orthology of *ABP19a*. Little is known about the function of *ABP19* in plant development, but a study found that *ABP19* can specifically bind to auxin in *Prunus persica*, and its activity or expression is regulated by auxin concentration [63]. Similarly, *ABP1* mediates cellular processes regulated by auxin and is affected by its concentration. At higher auxin concentrations, *ABP1* regulates cell growth and division, while at lower concentrations, it regulates cell expansion [35, 64]. During the development of *I. uliginosa*’s spurs, the correlation between auxin concentration and cellular processes aligns with the auxin response mediated by *ABP*, suggesting that endogenous hormones may regulate corresponding developmental processes by adjusting their content. As a crucial component in the auxin signaling pathway, *IuABP* likely plays a role not only in regulating cell division or elongation processes at various developmental stages but also in coordinating the transition between them [65]. A significant event during this transition is spur curve formation.

Silencing *IuABP* resulted in notable changes in spur morphology, characterized by an increase in the number of curves and a distortion in orientation. The cells on both sides of the *abp*_s spurs’ curves exhibit more significant numerical differences. We hypothesize that downregulating *IuABP* releases a signal resembling the decrease in auxin concentration in the WT spur, disrupting the spur’s normal growth and development pattern. This disruption leads to the premature and additional occurrence of the unbalanced event of proximal-distal cell division, which should have occurred only once during the middle stage. This regulation of cell division might be achieved by influencing cell cycle genes, as previously confirmed in studies of *Arabidopsis* [32, 66]. However, the coordination of cell programs seems to be important for *I. uliginosa* spur development. Downregulation of *IuABP* also leads to a greater degree of anisotropic elongation of cells on the curve that appears earlier. As we previously assumed, the formation of curves may initiate the process of cell elongation. Furthermore, the local inhibition of *ABP1* activity in tobacco shoot apical meristem causes cells to exhibit an irregular division pattern [32], which may explain why the curves of *abp*_s spurs form multi-angle distortion. The existing data provide us with the idea of the increasing number of *abp*_s spurs, but it is still insufficient to explain the cause. We will conduct more targeted additional experiments to explore the mechanism.

EXT, which belongs to the superfamily of plant cell wall proteins, affects cell wall morphology by adjusting its structure [67, 68]. Spur growth and development require active cell wall remodeling [25, 69]. The most significant phenotype caused by knocking down *IuEXT* is spur shortening. The length of strongly silenced spurs was only 5–6 mm, equivalent to the length of WT spurs at the early stage, indicating that cell elongation was almost completely contained. The significant decrease in cell elongation from the central to basal regions of *ext*_s spurs indicates that *IuEXT* positively regulates cell elongation in *I. uliginosa*, which is in contrast to the negative correlation between *EXT* ortholog and cell elongation in *Allium cepa*, *Oryza sativa*, and *Arabidopsis thaliana* [70–72]. These results indicate that *EXT* has a conservative and redundant function in regulating cell elongation. However, there may be differences in regulation patterns among different species due to the absence of fixed conserved domains in the *EXT* gene family [47].

Shortened *ext*_s spurs also lose their curves, suggesting that the proximal-distal cell division imbalance occurring during the middle stage is substantially reduced or eliminated. However, we suggest that *IuEXT* regulation on cell division affecting curves may not be direct; the failure of cell program conversion was due to anisotropic elongation inhibition, so the downstream genes controlling curve formation cannot be successfully started, showing an opposite effect on *abp*_s spurs. In addition, papilla formation can lead to changes in cell wall structure and morphology. Extensin is an important component of the primary plant cell wall, and the knockdown of *IuEXT* may affect the reconfiguration of cell walls, leading to abnormal papilla development. Trichomes (or papillae, hair) within spurs usually produce nectar and are thought to increase the total surface area for nectar secretion and reabsorption [25, 73, 74]. We hypothesize that the abnormal development of the inner epidermal papillae caused by *IuEXT* silencing may affect the nectar secretion of spurs, which will be confirmed by further nectar content and composition detection.

## Conclusion

In this study, we investigated spur development in *I. uliginosa* based on histomorphology and hormone levels, and the functions of *IuABP* and *IuEXT* were verified to explore the mechanism of spur development. Our results suggest that spurs reach their final length and morphology through cell division in the early stage and anisotropic elongation in the middle stage. Endogenous hormones regulate the developmental process of spurs by changing their concentrations. The expression of *IuABP* will affect the morphology, formation time, and number of spur curves. *IuEXT* regulates spur length by affecting the anisotropic elongation of spur cells, and it participates in the regulation of the development of the epidermal papillae within spurs.

## Materials and methods

### Plant materials and growth conditions

Seeds were collected from a wild *I. uliginosa* population in Laoyuhe National Wetland Park in Kunming and cultivated in a greenhouse of Southwest Forestry University. Growth conditions were maintained at 18°C–25°C, with 11–13 h of daylight and 40%–60% humidity.

### Scanning electron microscopy and histology

Tissues were fixed in FAA (50% ethanol, glacial acetic acid, and 38% formaldehyde at 18:1:1 ratio). The samples were dehydrated in an ethanol series, dried using an EMS (Hatfield, Pennsylvania, USA) 850 CO_2_ critical point dryer, and imaged using a ZEISS (Oberkochen, Germany) Sigma 300 SEM. Histological samples were dehydrated in an ethanol series, became transparent with xylene, waxed, and embedded. The tissues were sectioned with a thickness of 8 μm, stained with 0.01% Safranin O and 50% Fast Green [75], and imaged using a Leica (Wetzlar, Germany) DM750 optical microscope.

### Observation of the internal structure of flower buds and mature spurs

Flower buds of *I. uliginosa* were obtained from 10 WT plants and divided into five phases according to their size. The spurs cannot be observed with the naked eye for phases 1–3 buds. The spur length of the buds in phases 4 and 5 was 0.3 mm and 0.5 mm, respectively. The buds were observed by SEM and paraffin section. Mature spurs were obtained from WT and strongly *ext*_s plants, and the inner epidermal structure was observed by SEM after dissection. Due to the large volume of mature spurs, continuous photos were taken and merged through Photoshop.

### Cell counts and measurements

For WT plants, spurs at 2 mm (stage 1, early stage), 4 mm (stage 2, early stage), 9 mm (stage 3, middle stage), 13 mm (stage 4, middle stage), and 22 mm (stage 5, anthesis stage) were selected (Figs S7A). Three biological replicates (spur) were selected from 2 or 3 plants at each stage. For *abp*_s plants, spurs at the 9-mm stage were selected because the twisting curves were most obvious. For *ext*_s plants, spurs at the anthesis stage were selected. Three biological replicates (spur) were selected from two or three silenced plants. Longitudinal paraffin sections of spurs were made, and complete images were obtained using a CaseViewer for cell counts; a measurement tool was used to obtain the spur length data. The epidermal cells on the proximal side of WT and *ext*_s spurs were counted from the base to the tip. The proximal-distal epidermal cells at the curved part at the 9 mm stage (WT and abp_s spurs, about 1.3-mm long range, 90° angle) and the anthesis stage (WT spurs, about 5.2-mm long range, 30° angle) were counted (Fig. S7B, see online supplementary material) [17, 19]. In addition, SEM was used to randomly select two to three visual fields of the base, middle, and tip of WT and *ext*_s spurs, as well as the proximal and distal sides of the curved part of *abp*_s spurs (9 mm stage) and WT spurs (9 mm stage and the anthesis stage). Ten cells were randomly selected from these two to three fields for measurement (Fig S7B and C, see online supplementary material). ImageJ was used to measure the maximum cell length and width [19]. Cell anisotropy was calculated based on the ratio of cell length to width [22].

### Hormone content determination

The labellum was removed from WT flowers with spur lengths of 5–8 mm (early stage) and 13–16 mm (middle stage). The spur and limb tissues were frozen separately with liquid nitrogen (see Fig. S7C, see online supplementary material). We collected mixed samples from over 100 plants, each sample with three biological replicates, each weighing at least 100 mg. After crushing, the samples were extracted and purified. The extracts were then analysed using a Vanquish UPLC-Orbitrap-MS system (ThermoFisher, Waltham, MA, USA). HRMS data were recorded on a Q Exactive hybrid Q-Orbitrap mass spectrometer (ThermoFisher, Waltham, MA, USA) using SIM MS acquisition methods, and data were processed using TraceFinder [76, 77].

### Isolation and identification of candidate genes

Sequences and annotation information of *IuABP* (TRINITY_DN6970_c0_g1) and *IuEXT* (TRINITY_DN9685_c0_g1) were obtained from previous transcriptome data [31]. Total RNA was extracted from labella using an Omega (Norcross, Georgia, USA) E.Z.N.A Plant RNA Kit. cDNA was obtained by reverse transcription using Transgen (Beijing, China) EasyScript One-Step gDNA Removal and cDNA Synthesis SuperMix, which was used as a template to amplify the cDNA sequences of *IuABP* and *IuEXT*. Amplified fragments were purified and cloned into a TaKaRa (Dalian, China) pMDTM19-T Vector for sequencing. Primers used for gene isolation are listed in Table S2 (see online supplementary material). To confirm the orthology of *IuABP* and *IuEXT*, coding sequences of homologous genes from other species were obtained through BLAST search on GenBank. Among them, the 20 members of classical extensin in *Arabidopsis* were identified by Showalter *et al.* [67]. Phylogenetic analysis was performed in MEGA-X using the maximum-likelihood method (Figs S1 and S2, see online supplementary material) [78]. All DNA sequences were aligned using Clustalw [79], and 1000 bootstrap replicates were performed.

### Virus-induced gene silencing

A 309-bp fragment of *IuABP* and a 165-bp fragment of *IuEXT* were amplified using primers that incorporated XbaI and BamHI sites at the 5′ and 3′ ends, respectively (Table S2, see online supplementary material). The fragments were introduced into a tobacco rattle virus 2 (TRV2) vector to generate TRV2-*IuABP* and TRV2-*IuEXT* constructs. These constructs were transformed into GV3101 *Agrobacterium* cells by the freeze–thaw method [80, 81]. Then, 75 flower branches from 15 plants were treated with a TRV2-*IuABP* construct, 110 flower branches from 22 plants were treated with a TRV2-*IuEXT* construct, while 56 flower branches from 10 plants were treated with a TRV2 construct (empty vector). Each flower branch contains 8–30 flower buds. Flowers with silencing phenotypes were photographed, and their spur lengths were measured. The spurs were frozen at −80°C for subsequent RNA expression analysis or fixed in FAA solution for histological anatomy and SEM analysis.

### Quantitative real-time polymerase chain reaction

qRT-PCR experiments were conducted to investigate the silencing efficiency of VIGS experiments. Total RNA was extracted from WT, TRV2 (empty vector), *IuABP*-silenced, and *IuEXT*-silenced tissues; then, it was reverse transcribed. The obtained cDNA was diluted 10 times and used as templates. qRT-PCR was performed on Roche (Rotkreuz, Switzerland) LightCycler 480II Real-Time Quantitative PCR Detection System using Hieff (Shanghai, China) qPCR SYBR Green Master Mix. Relative gene expression values were calculated using a comparative CT (2^−ΔΔCT^) method [82]. *IuActin* was used as an internal control. The primers used are listed in Table S2 (see online supplementary material). There were three biological replicates per sample, each with three technical replicates.

#### Accession numbers

Sequence data from this article can be found in the GenBank under accession numbers *IuABP* (ON803510.1) and *IuEXT* (OR036915).

## Supplementary Material

Web_Material_uhae015
