# Supplementary material for: The cellular and molecular basis of the spur development in Impatiens uliginosa
Source: Hortic Res. 2024 Jan 12;11(3):uhae015. doi: 10.1093/hr/uhae015 (PMC10967693; doi:10.1093/hr/uhae015)
Supplement: Web_Material_uhae015 [file web_material_uhae015.zip › Supplemental Material.docx]

**
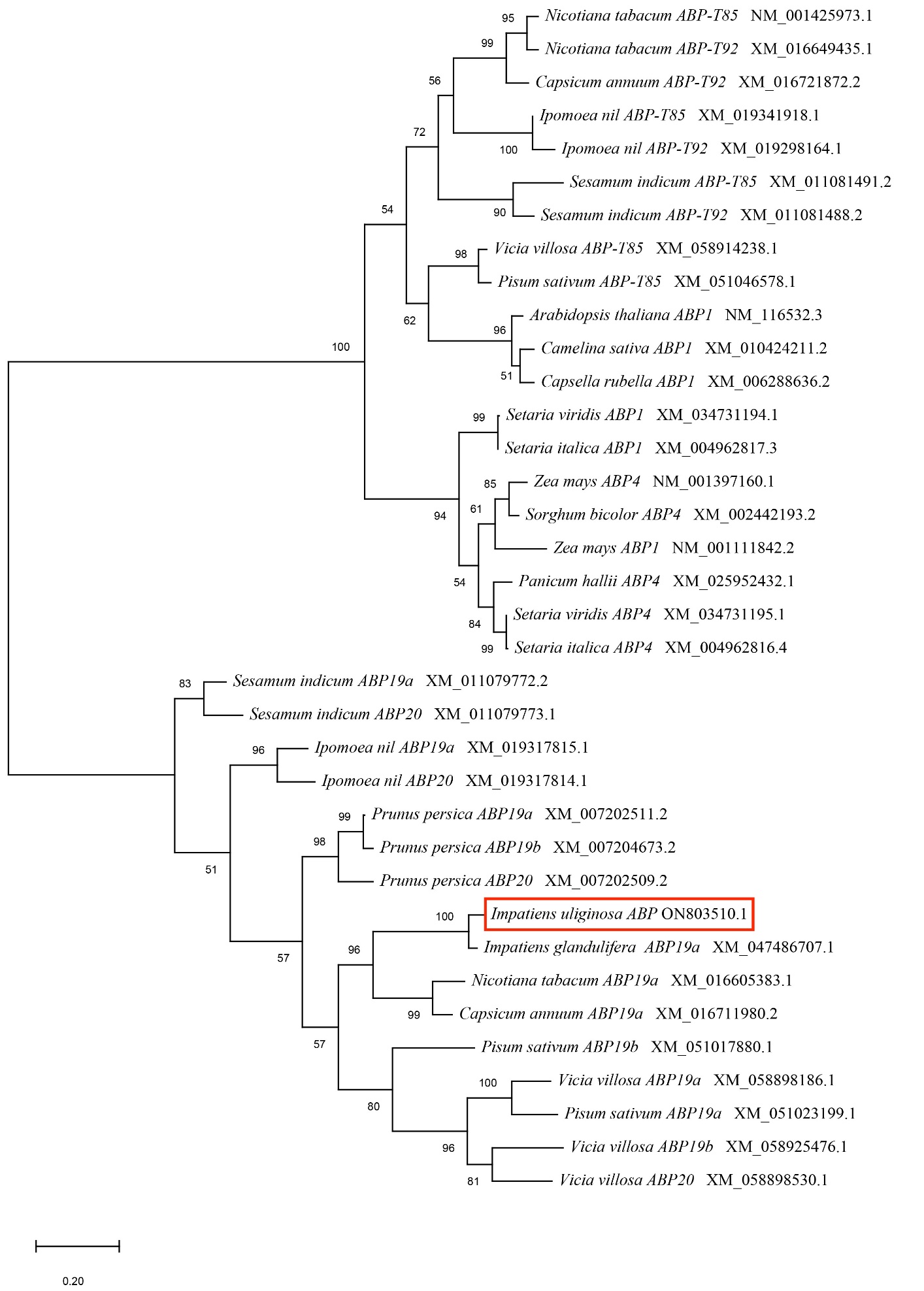
**

**Figure S1. Phylogenetic tree of *ABP* genes.** The phylogenetic tree was constructed using the maximum-likelihood method. The tree is divided into two distinct clades, the first containing *ABP1*, *ABP4,* *ABP-T85*, and *ABP-T92*, and the second containing *ABP19a*, *ABP 19b*, and *ABP20*. *ABP* genes from monocotyledonous plants are clustered in the first clade. The gene investigated in this study is shown in the red box. Partial gene identifiers are as follows: ***Arabidopsis thaliana ABP1***: AT4G02980; ***Capsella rubella ABP1***: CARUB_v10002006mg; ***Zea mays ABP1***: ZEAMMB73_Zm00001d041711; ***Setaria viridis ABP1***: SEVIR_3G364800v2; ***Setaria italica ABP1***: SETIT_024732mg; ***Zea mays ABP4***: ZEAMMB73_Zm00001d023908; ***Sorghum bicolor ABP4***: SORBI_3008G113700; ***Panicum hallii ABP4***: PAHAL_3G448500; ***Setaria viridis ABP4***: SEVIR_3G364700v2; ***Setaria italica ABP4***: SETIT_024187mg; ***Pisum sativum ABP19a***: KIW84_052746; ***Pisum sativum ABP19b***: KIW84_045735; ***Pisum sativum ABP T85***: KIW84_UN0606; ***Prunus persica ABP19a***: PRUPE_7G200300; ***Prunus persica ABP19b***: PRUPE_7G200200; ***Prunus persica ABP20***: PRUPE_7G200100.

**
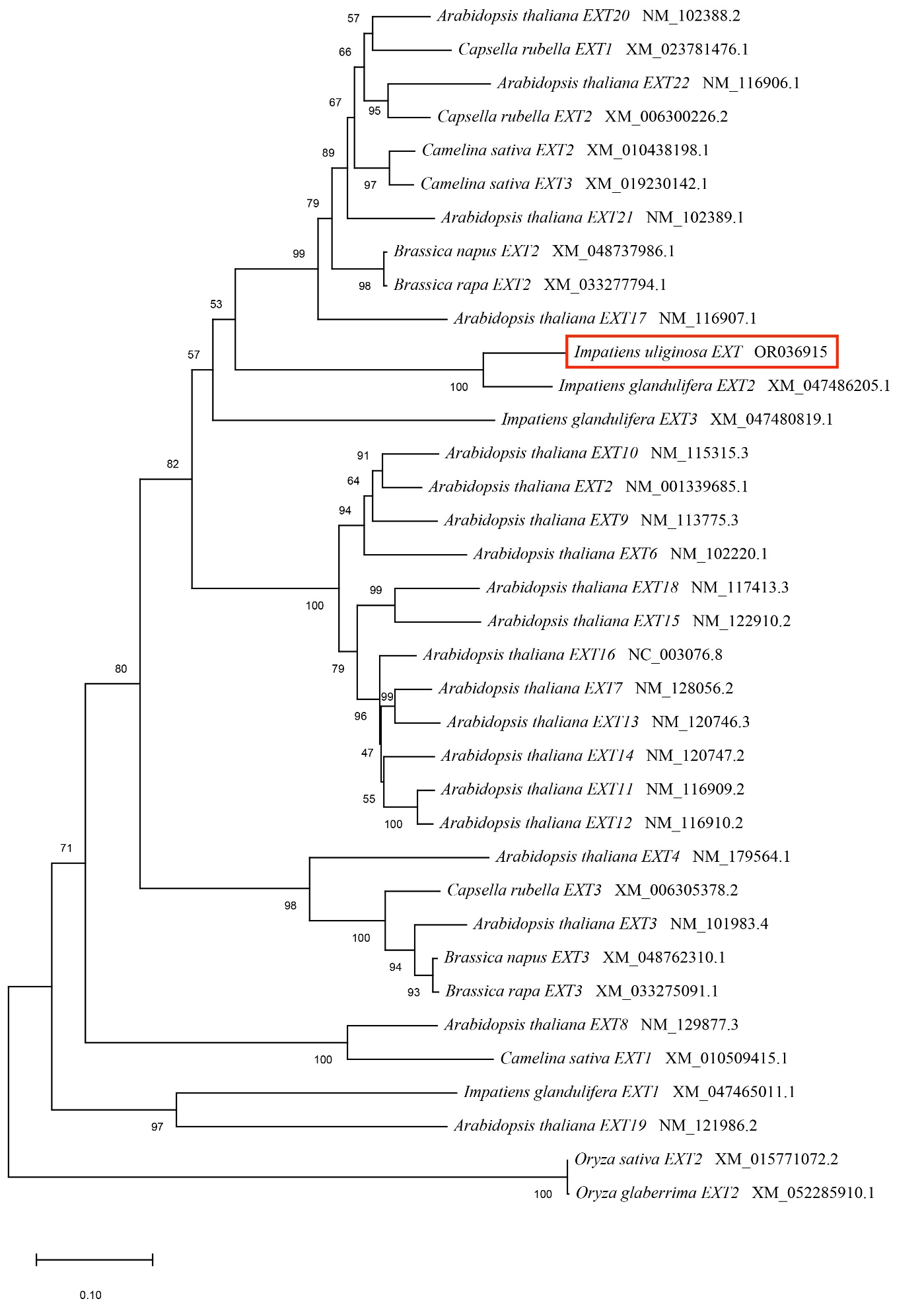
**

**Figure S2. Phylogenetic tree of *EXT* genes.** The phylogenetic tree was constructed by the maximum-likelihood method. The two monocotyledonous plants at the bottom of the tree are outgroup. The gene investigated in this study is shown in the red box. Partial gene identifiers are as follows: ***Arabidopsis thaliana EXT2***: At3g54590; ***Arabidopsis thaliana EXT3***: At1g21310; ***Arabidopsis thaliana EXT4***: At1g76930; ***Arabidopsis thaliana EXT6***: At1g23720; ***Arabidopsis thaliana EXT7***: At2g24980; ***Arabidopsis thaliana EXT8***: At2g43150; ***Arabidopsis thaliana EXT9***: At3g28550; ***Arabidopsis thaliana EXT10***: At3g54580; ***Arabidopsis thaliana EXT11***: At4g08400; ***Arabidopsis thaliana EXT12***: At4g08410; ***Arabidopsis thaliana EXT13***: At5g06630; ***Arabidopsis thaliana EXT14***: At5g06640; ***Arabidopsis thaliana EXT15***: At5g35190; ***Arabidopsis thaliana EXT16***: At5g49080; ***Arabidopsis thaliana EXT17***: At4g08380; ***Arabidopsis thaliana EXT18***: At4g13390; ***Arabidopsis thaliana EXT19***: At5g19810; ***Arabidopsis thaliana EXT20***: At1g26240; ***Arabidopsis thaliana EXT21***: At1g26250; ***Arabidopsis thaliana EXT22***: At4g08370; ***Oryza sativa EXT2***: OSNPB_020208900; ***Capsella rubella EXT2***: CARUB_v100161970mg; ***Capsella rubella EXT3***: CARUB_v10009850mg; ***Brassica napus EXT2***: HID58_030534; ***Brassica napus EXT3***: HID58_075717.

**
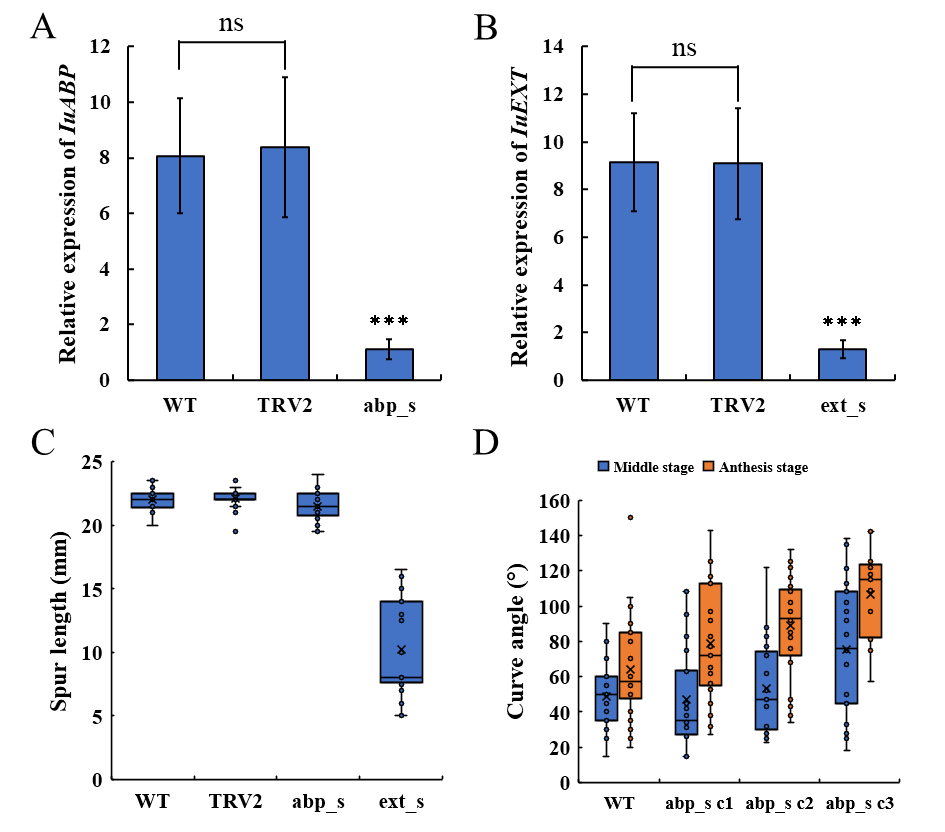
**

**Figure S3. Expression analysis of silenced spurs and statistics of their length and angle.** (A) Relative expression levels of IuABP in WT, TRV2, and *abp_s* spurs. (B) Relative expression levels of IuEXT in WT, TRV2, and *ext_s* spurs. (C) Length of WT (n = 30), TRV2 (n = 25), *abp_s* (n = 25), and *ext_s* (n = 24) spurs. (D) Angle of WT (n = 30) and three curves of *abp_s* (n = 25) spurs. Asterisks indicate a significant difference relative to WT spurs (T-Test: ***, P < 0.001; ns, not significant). c1, c2, and c3 in (D) indicate curve1, curve2, and curve3 of *abp_s* spurs, respectively.

**
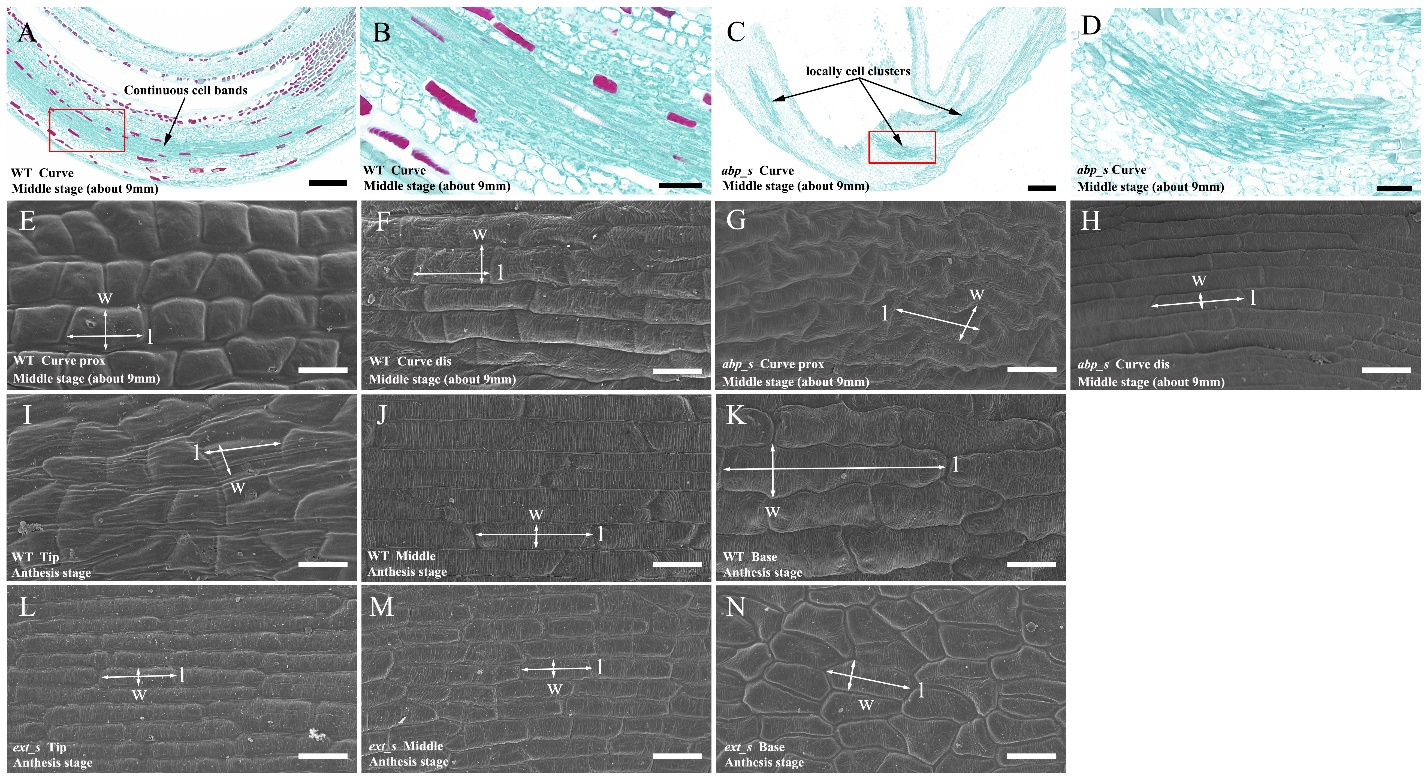
**

**Figure S4. Cell morphology of WT, *abp_s*, and *ext*_s spurs.** (A) Longitudinal anatomy of the WT spur’s curved part in the middle stage (about 9 mm). In the distal region of spurs, several layers of tightly arranged parenchymal cells subjected to bending mechanical stress appear as a complete, continuous cell band. (B) Partial magnification of the red box area in A. (C) Longitudinal anatomy of *abp*_s spur’s curved part in the middle stage (about 9 mm). The cell band breaks due to the twisting of spurs, forming locally squeezed cell clusters. (D) Partial magnification of the red box area in C. (E-F) Cell morphology at the proximal (prox) (E) and distal (dis) (F) side of WT spur’s curve in the middle stage (about 9 mm). (G-H) Cell morphology at the proximal (prox) (G) and distal (dis) (H) side of *abp*_s spur’s curve in the middle stage (about 9 mm). (I-K) Cell morphology at the tip (I), middle (J), and base (K) of WT spurs at the anthesis stage. (L-N) Cell morphology at the tip (L), middle (M), and base (N) of *ext*_s spurs at the anthesis stage. Horizontal and vertical lines indicate cell length (l) and width (w). Bars = 200 μm in (A) and (C). Bars = 50 μm in (B) and (D). Bars = 20 μm in (E-N).

**
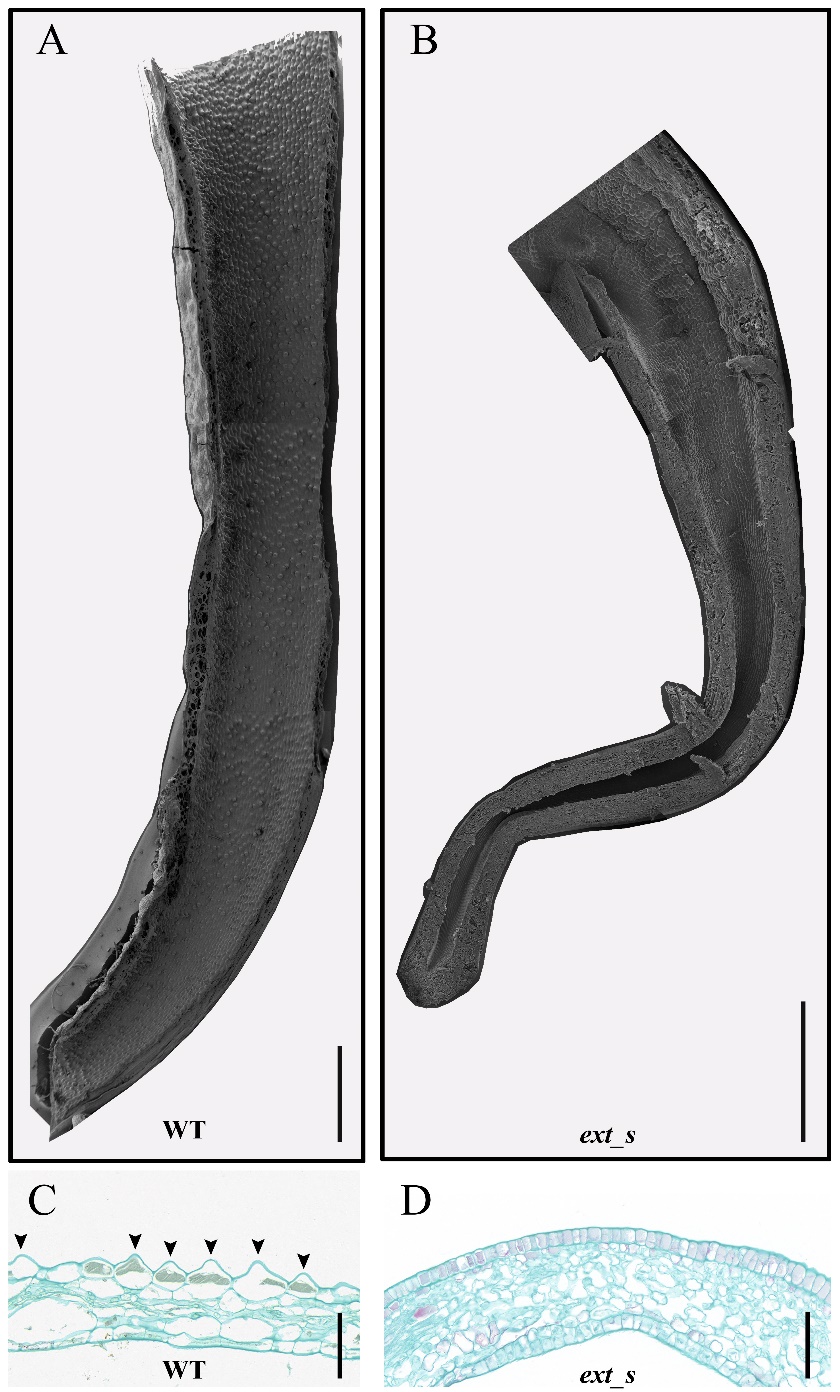
**

**Figure S5. Inner epidermal structure of WT and *ext*_s mature spurs.** (A) The inner epidermal cells of WT mature spurs have papillae, and the degree of protrusion of the papillae decreased gradually from the base to the tip. (B) Most inner epidermal cells of *ext*_s spurs have lost their papillae, and only a few cells have a slight protrusion. (C) Histological section of mature WT spurs. (D) Histological section of ext_s spurs. The arrows indicate the papillae of the inner epidermal cells. Bars = 1 mm in (A-B). Bars = 100 μm in (C–D).


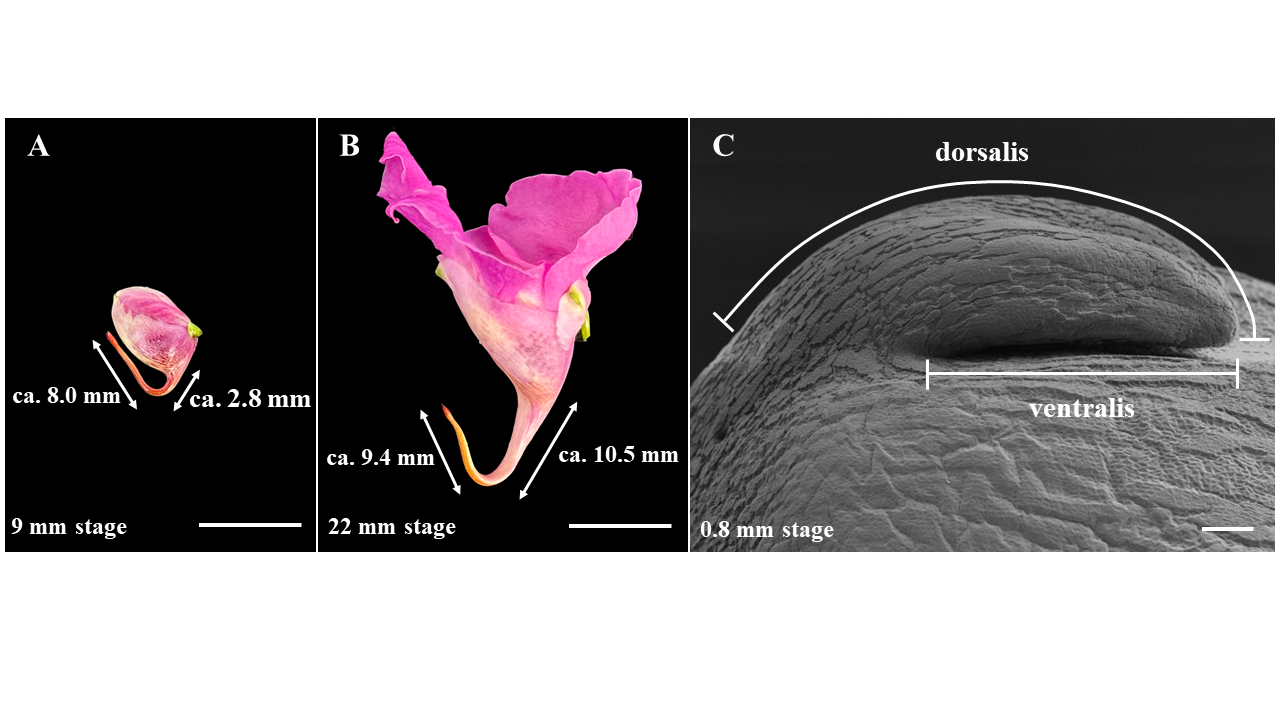


**Figure S6. *I. uliginosa* at the 0.8****-mm stage, 9-mm stage, and 22-mm stage.** (A) 9-mm spur; the length from the curve to the base is much less than that from the curve to the tip. (B) 22-mm spur; the length from the curve to the base increased significantly compared to that of the 9-mm stage, while the length from the curve to the tip slightly increased. (C) Nascent spur; differences in cell division result in a bigger dorsal length than the ventral length, leading to the spur growing toward the top of the bud. Bars = 10 mm in (A-B). Bars = 100 μm in (C).


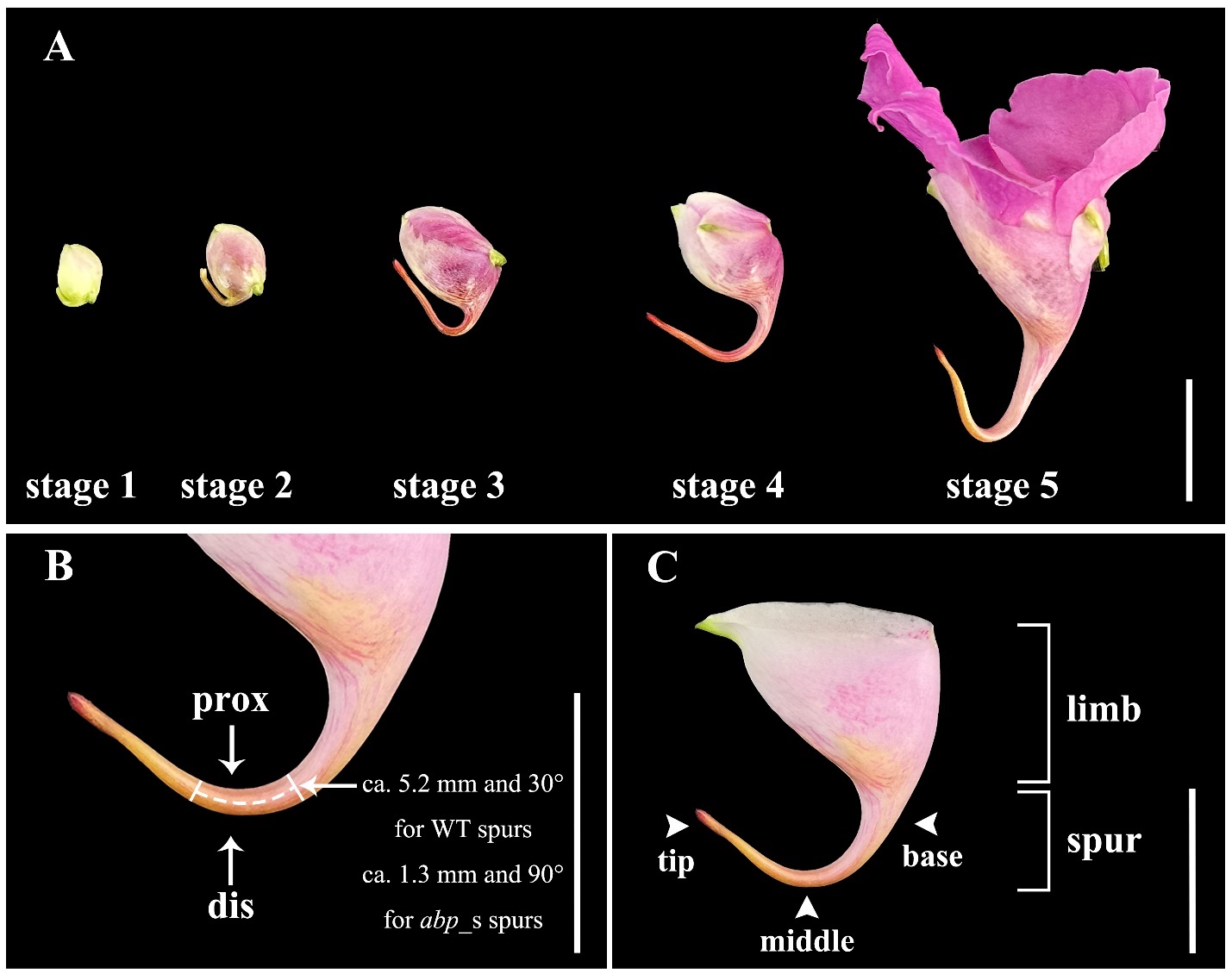


**Figure S7.** **Developmental stages and tissue sites of spurs for cell counting and measurement.** (A) Five developmental stages of spurs for cell counting and measurement. Spur length: stage 1, 2 mm (early stage); stage 2, 4 mm (early stage); stage 3, 9 mm (middle stage); stage 4, 13 mm (middle stage); stage 5, 22 mm (anthesis stage). (B) Proximal (prox) and distal (dis) part of the spur curve. (C) The labellum of *I. uliginosa* consists of spur and limb. Cells were selected for measurement at the spur base, middle, and tip, as shown by the arrows. Bars = 10 mm.

**Table S1: List of average spur length, cell number, and morphological indicators (cell length, cell width, and cell anisotropy) for five developmental stages**

| **Developmental stage** | | **Stage1** | **Stage2** | **Stage3** | **Stage4** | **Stage5** |
| --- | --- | --- | --- | --- | --- | --- |
| **Average spur length (mm)** | | 2.32 | 3.99 | 8.96 | 13.26 | 21.51 |
| **Average cell number** | | 213.33 | 272.33 | 340.67 | 340.67 | 341.67 |
| **Average cell length (μm)** | **Tip** | 16.89 | 20.03 | 25.63 | 26.73 | 28.42 |
|  | **Middle** | 13.70 | 17.75 | 34.97 | 40.02 | 60.21 |
|  | **Base** | 17.13 | 16.59 | 36.39 | 48.07 | 73.83 |
| **Average cell width (μm)** | **Tip** | 9.93 | 10.53 | 9.94 | 12.10 | 12.24 |
|  | **Middle** | 7.82 | 7.42 | 10.12 | 10.71 | 9.74 |
|  | **Base** | 7.16 | 7.48 | 10.16 | 11.35 | 14.63 |
| **Average cell antisotropy (L/W)** | **Tip** | 1.75 | 1.97 | 2.65 | 2.25 | 2.43 |
|  | **Middle** | 1.84 | 2.49 | 3.52 | 3.80 | 6.26 |
|  | **Base** | 2.37 | 2.28 | 3.67 | 4.30 | 5.21 |

All values in the table are rounded to two decimal places.

**Table S2: List of primers**

| **Gene** | **Forward primer (5’-3’)** | **Reverse primer (5’-3’)** |
| --- | --- | --- |
| **RT-PCR** | | |
| *IuABP* | ATGTTGCGCCTCGTTTTC | TTAATTGGTTCCTCCAAGAACACC |
| *IuEXT* | ATGGGAATCATCACTCAAGG | TTAATAGTGGTAAGGTGGAGGAG |
| **VIGS** | | |
| *IuABP* | TCTAGAACCGAGGTTTTGCTTG | GGATCCATTGGTTCCTCCAAG |
| *IuEXT* | TCTAGAGTTTACAAATCTCCTCCACC | GGATCCTGGATTGTATGGTTTTGTG |
| **qRT-PCR** | | |
| *IuABP* | CGGGCTTTGTGGCTCAATAC | TTCGCAAACAGCGCGAAATC |
| *IuEXT* | CCATTACAAATCACCTCCCCC | TGGATTGTATGGTTTTGTGGGC |

The underlines represent the restriction enzyme sites.
