# Supplementary figures and images for: The cellular and molecular basis of the spur development in Impatiens uliginosa
Source: Hortic Res. 2024 Jan 12;11(3):uhae015. doi: 10.1093/hr/uhae015 (PMC10967693; doi:10.1093/hr/uhae015)

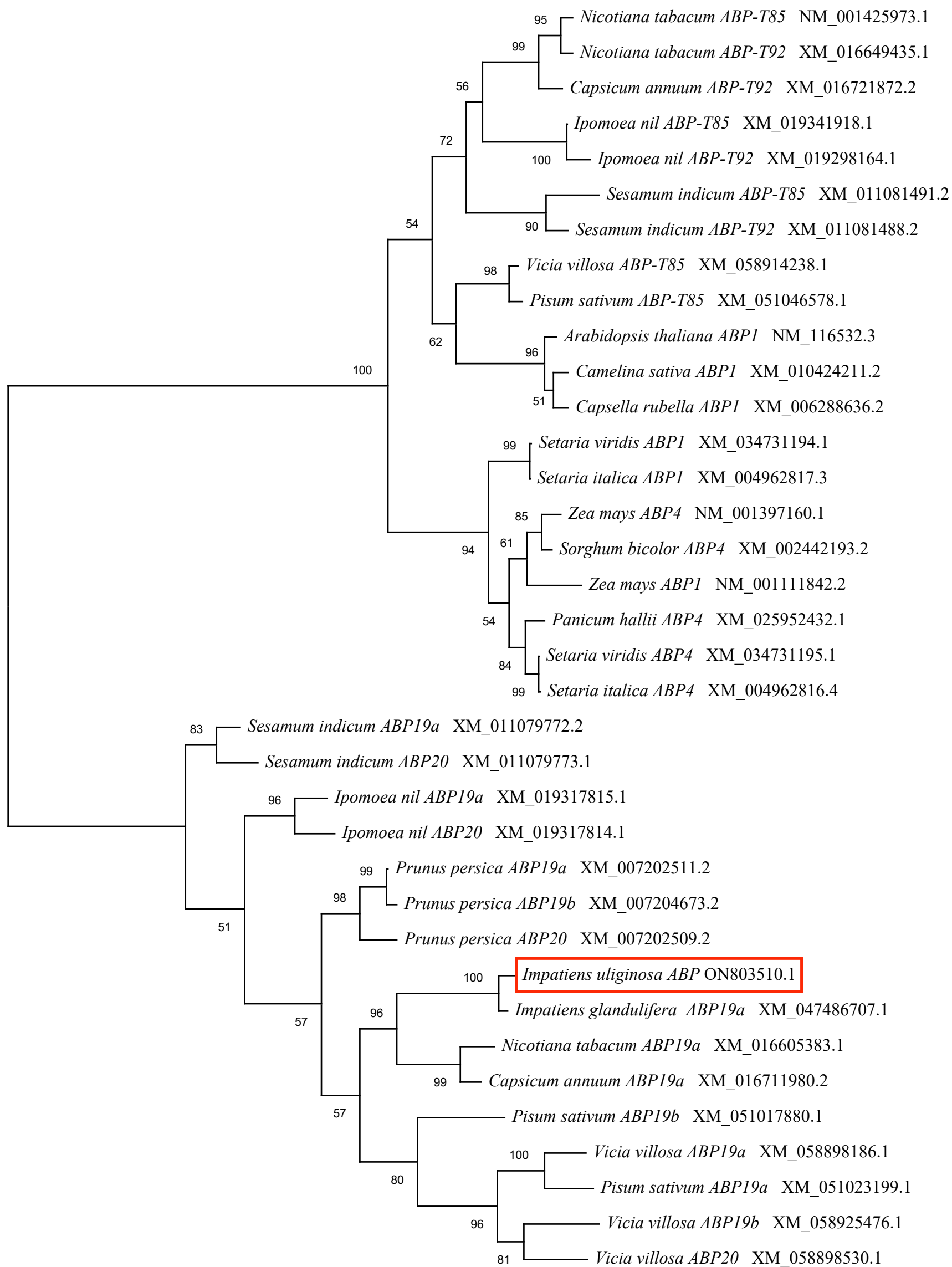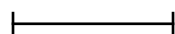

0.20

Supplement: Web_Material_uhae015 [file web_material_uhae015.zip › Fig S1.pdf]

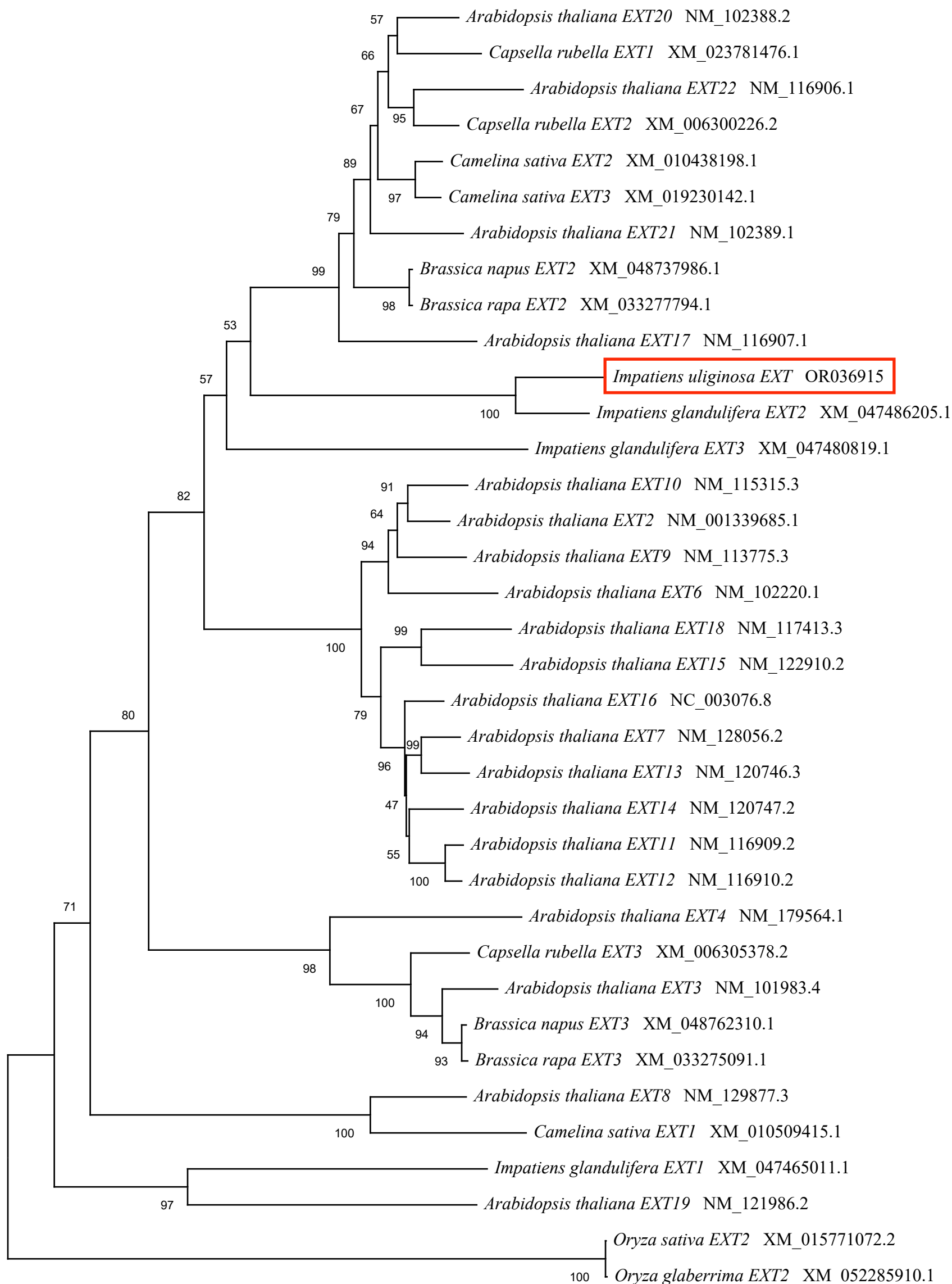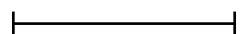

0.10

Supplement: Web_Material_uhae015 [file web_material_uhae015.zip › Fig S2.pdf]

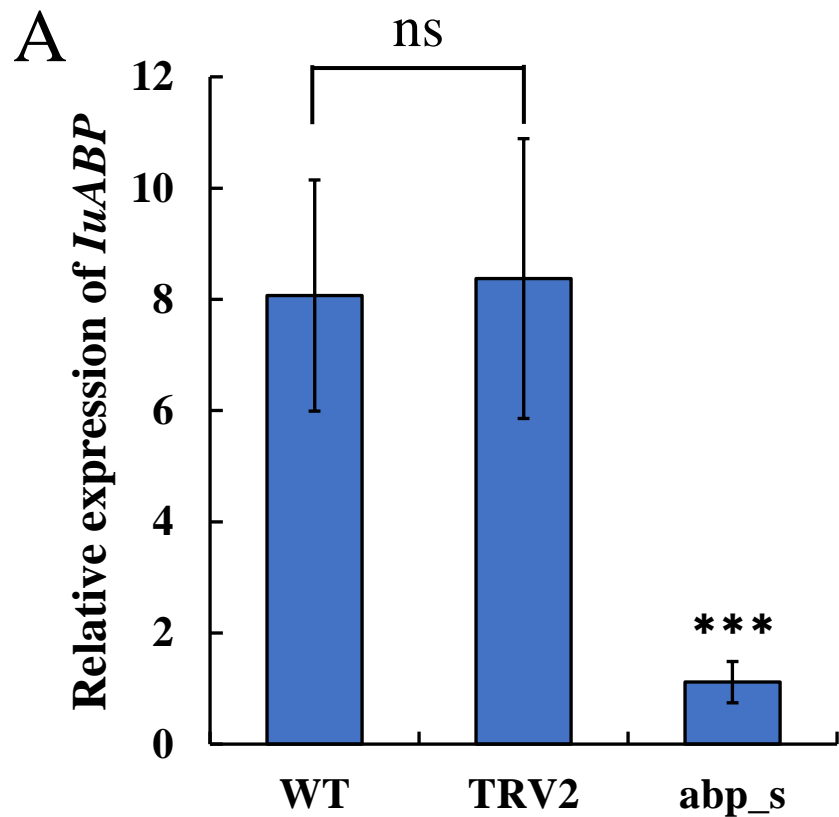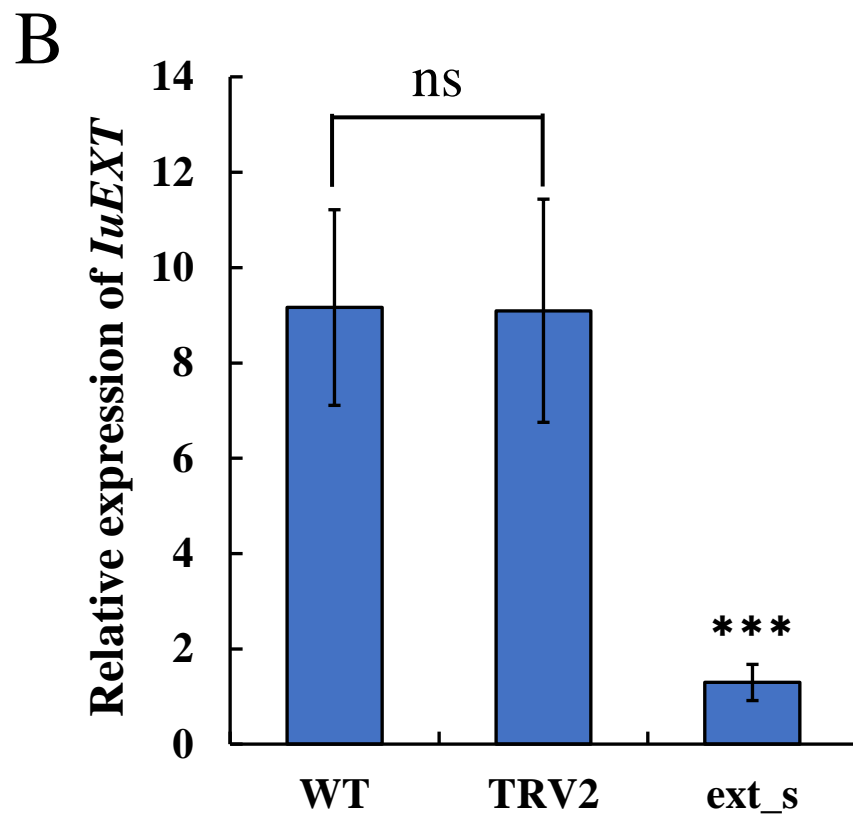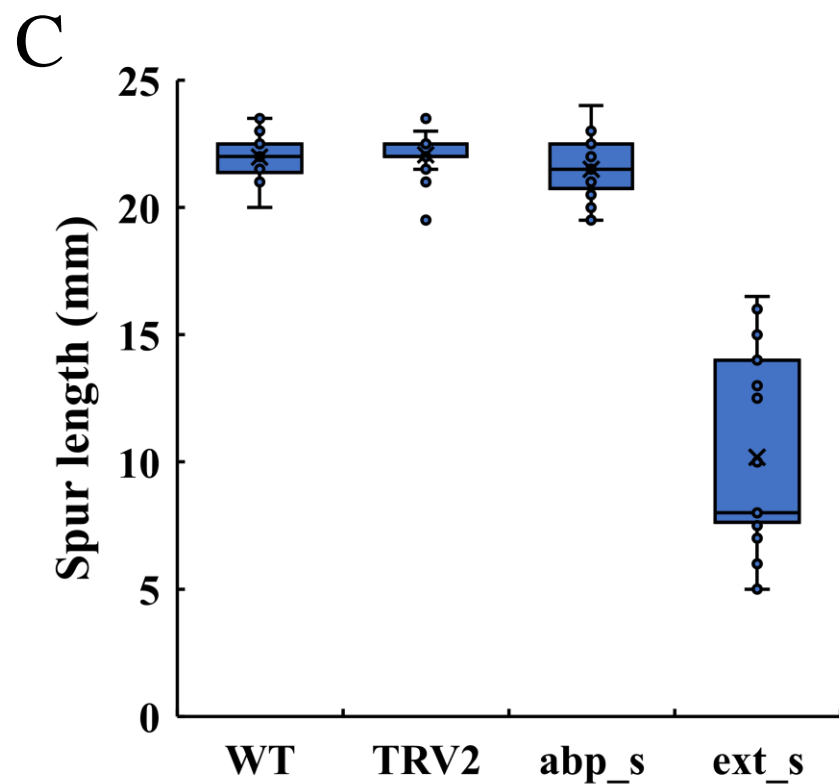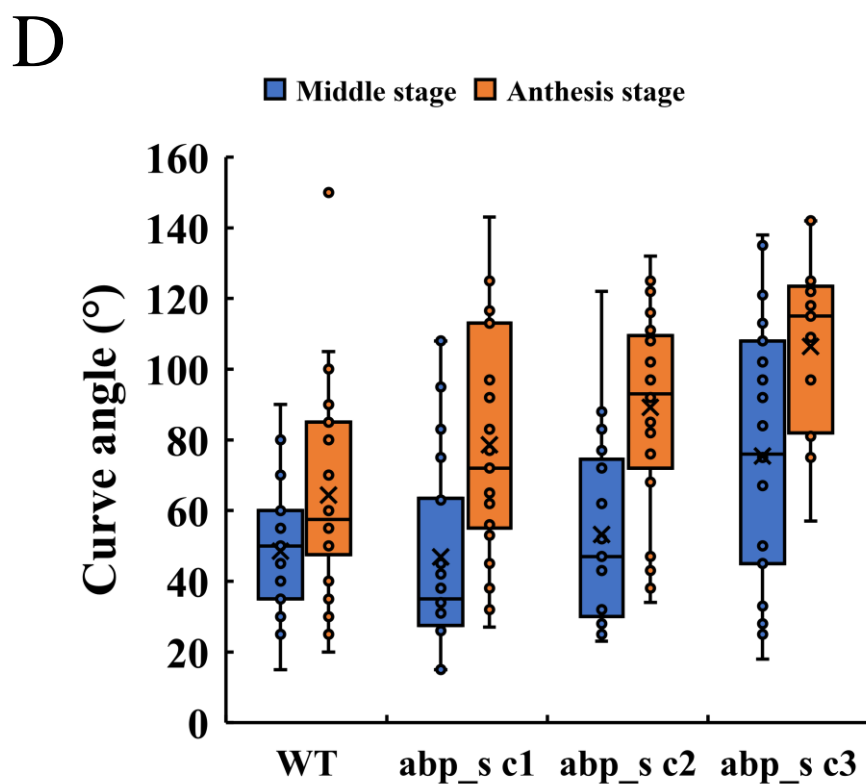

Supplement: Web_Material_uhae015 [file web_material_uhae015.zip › Fig S3.pdf]

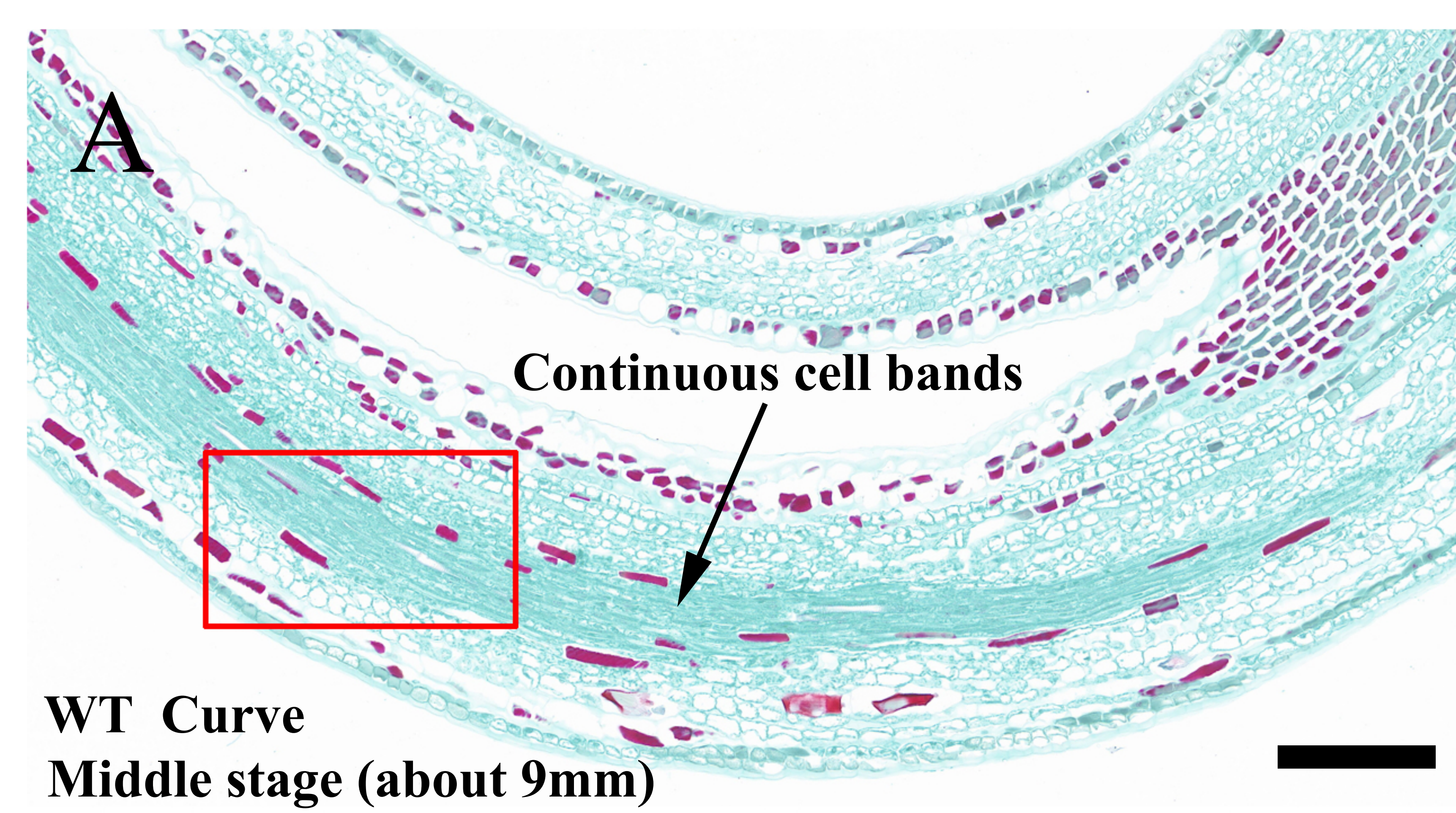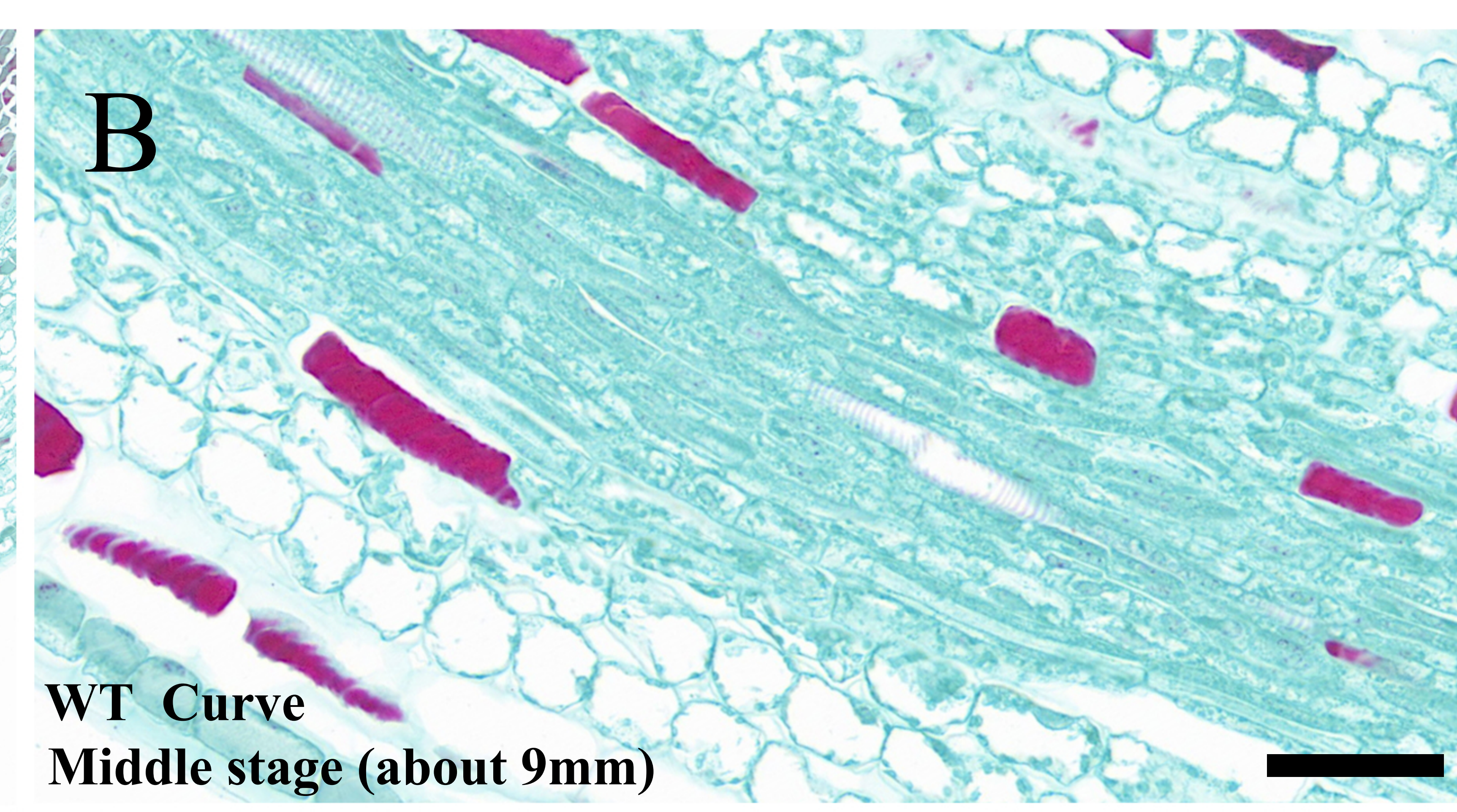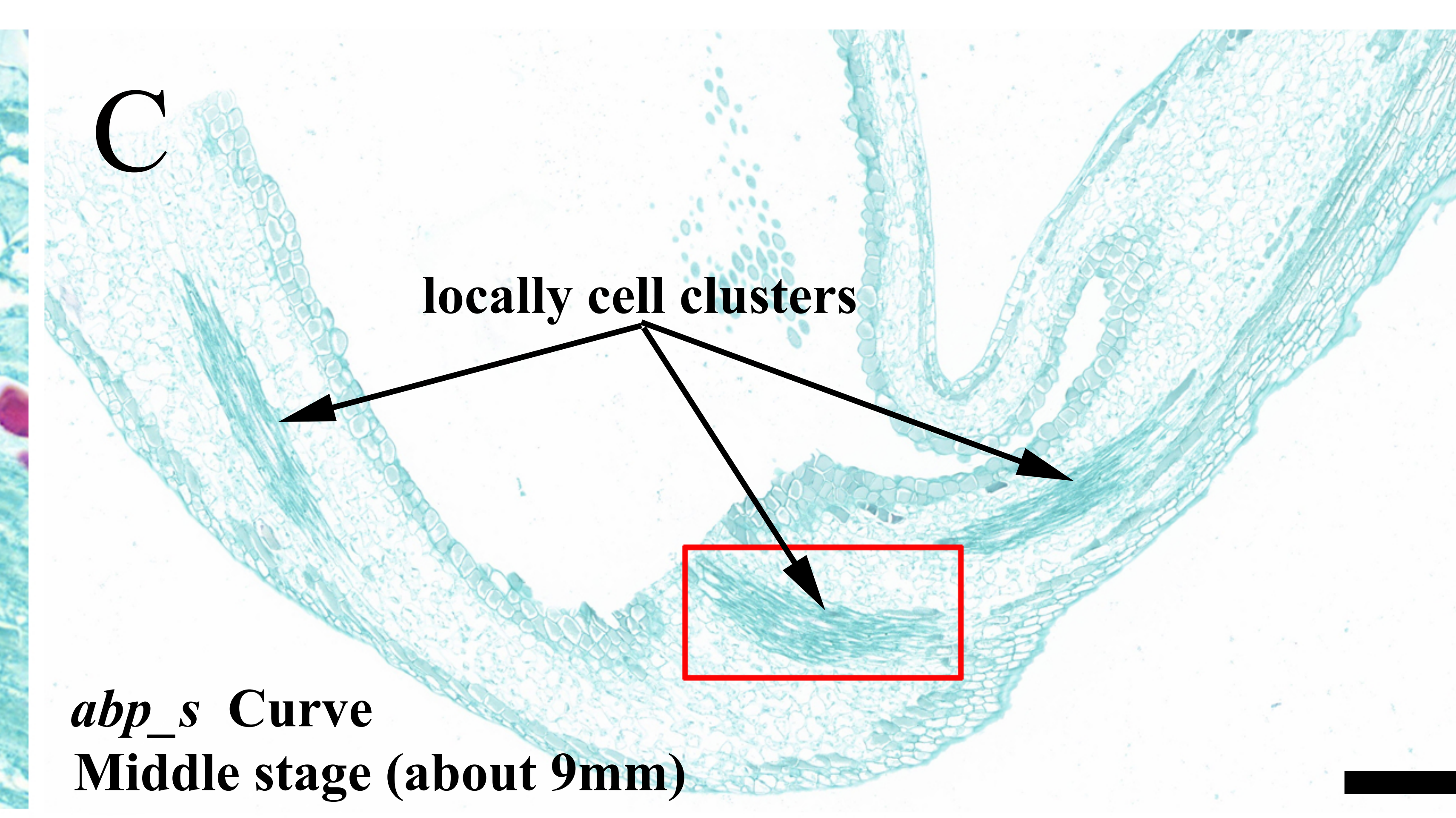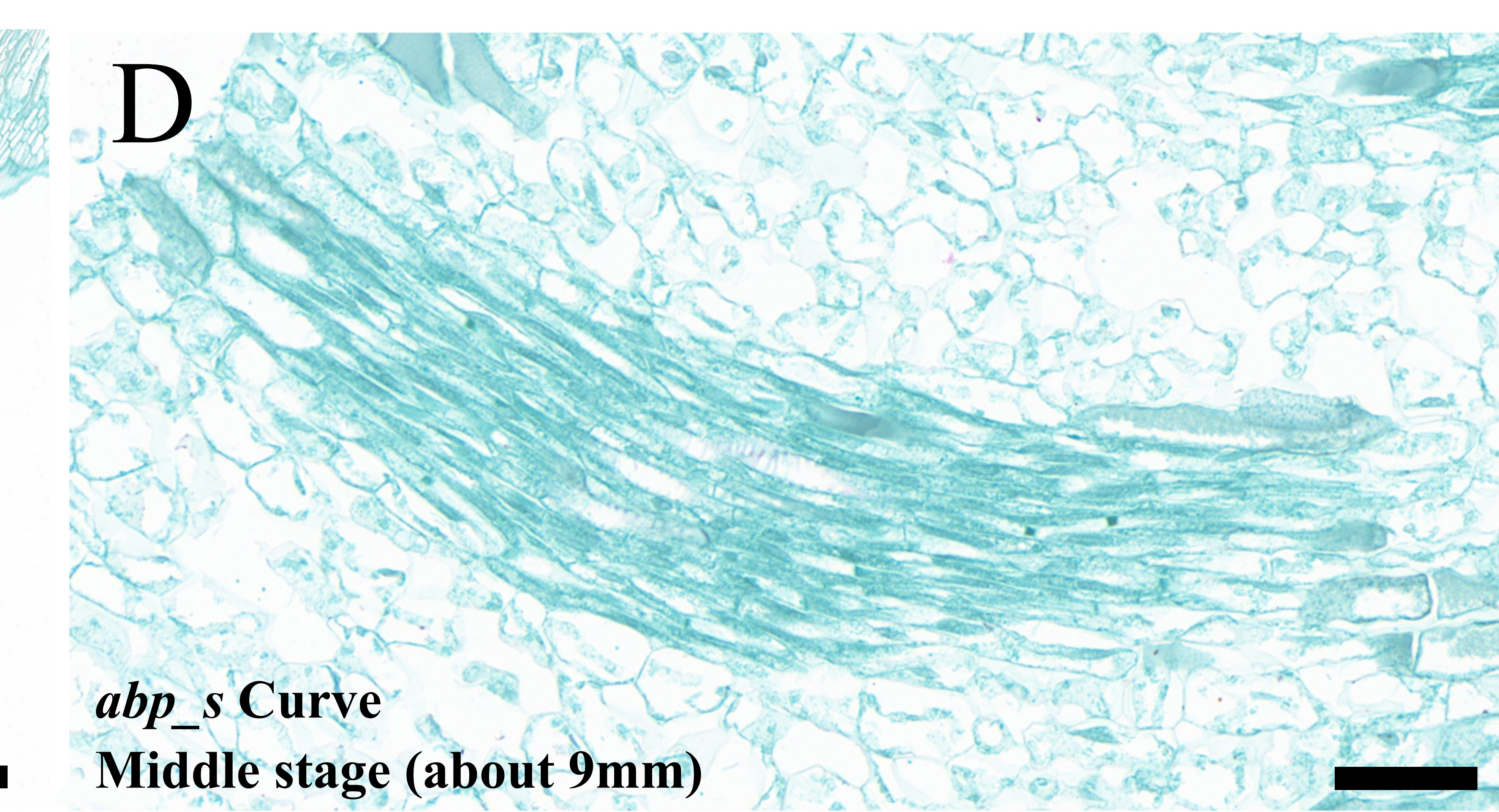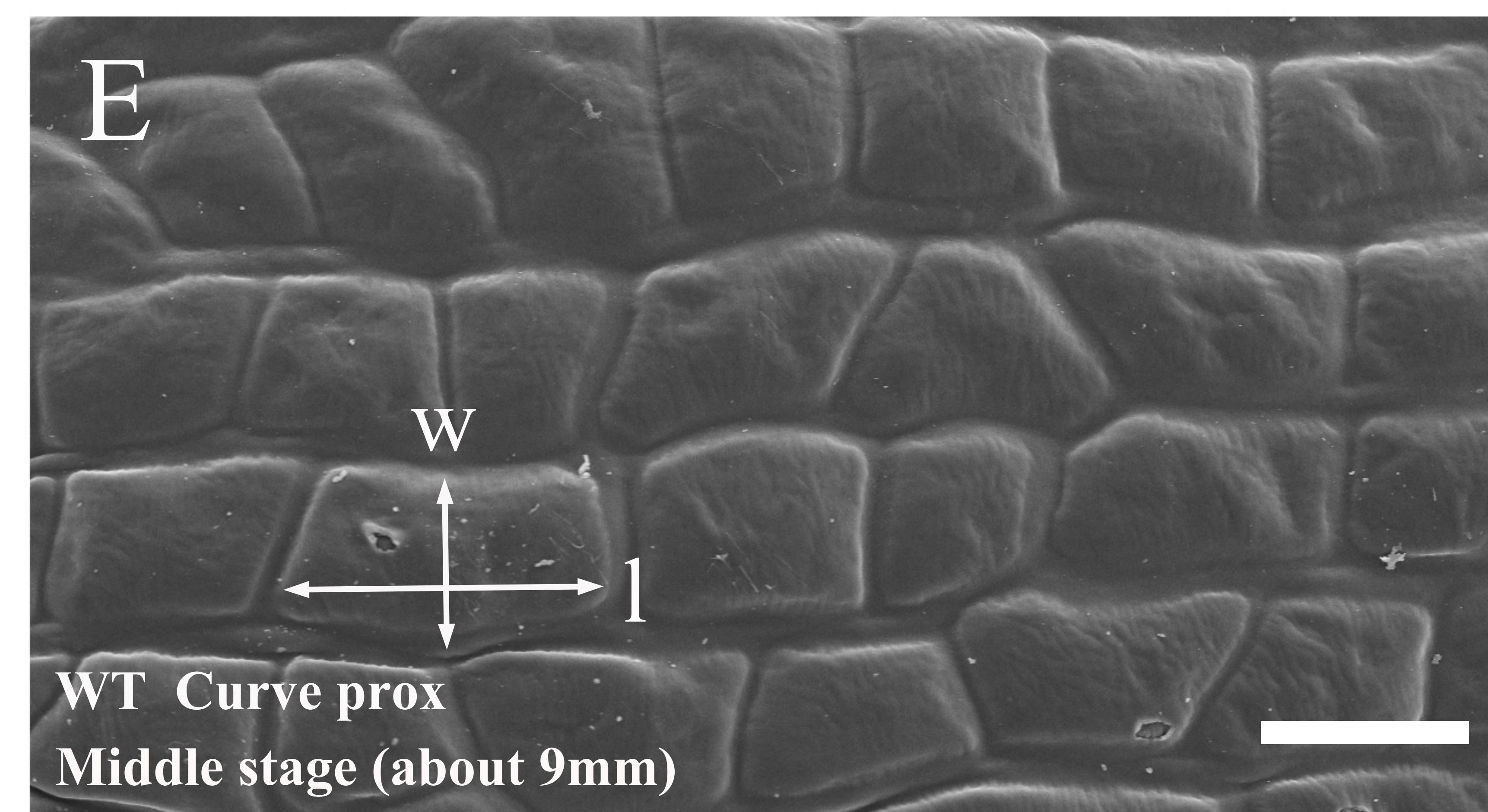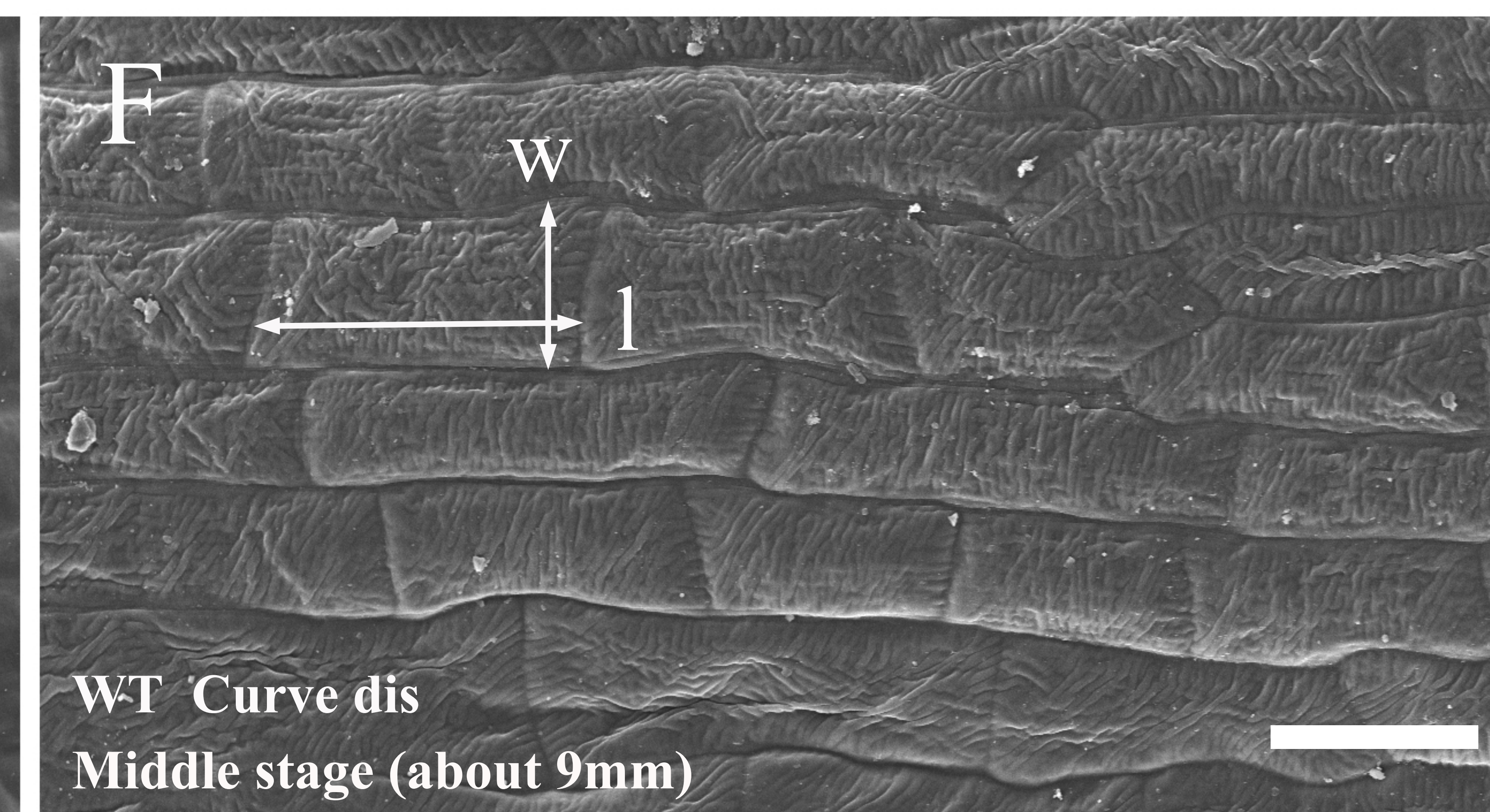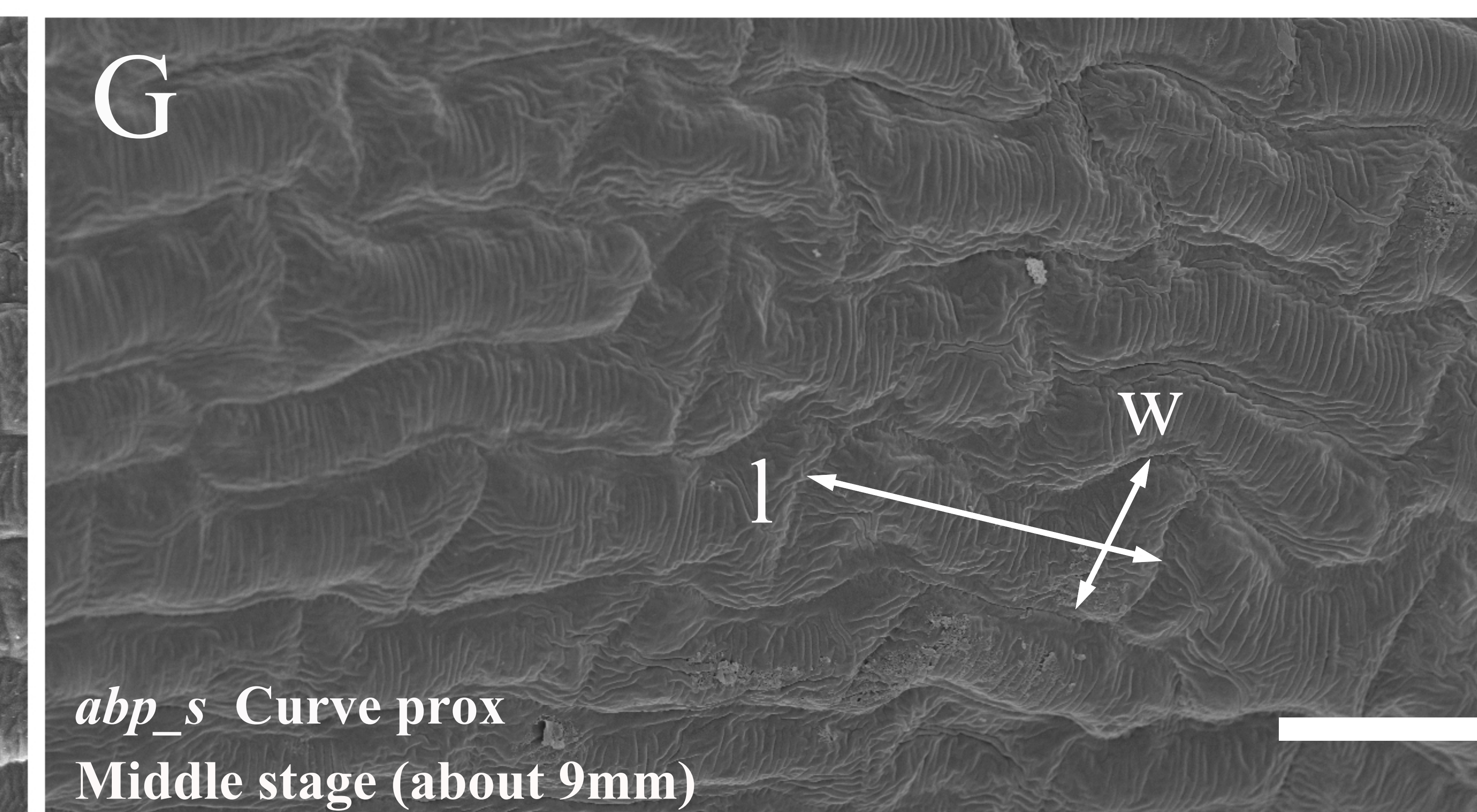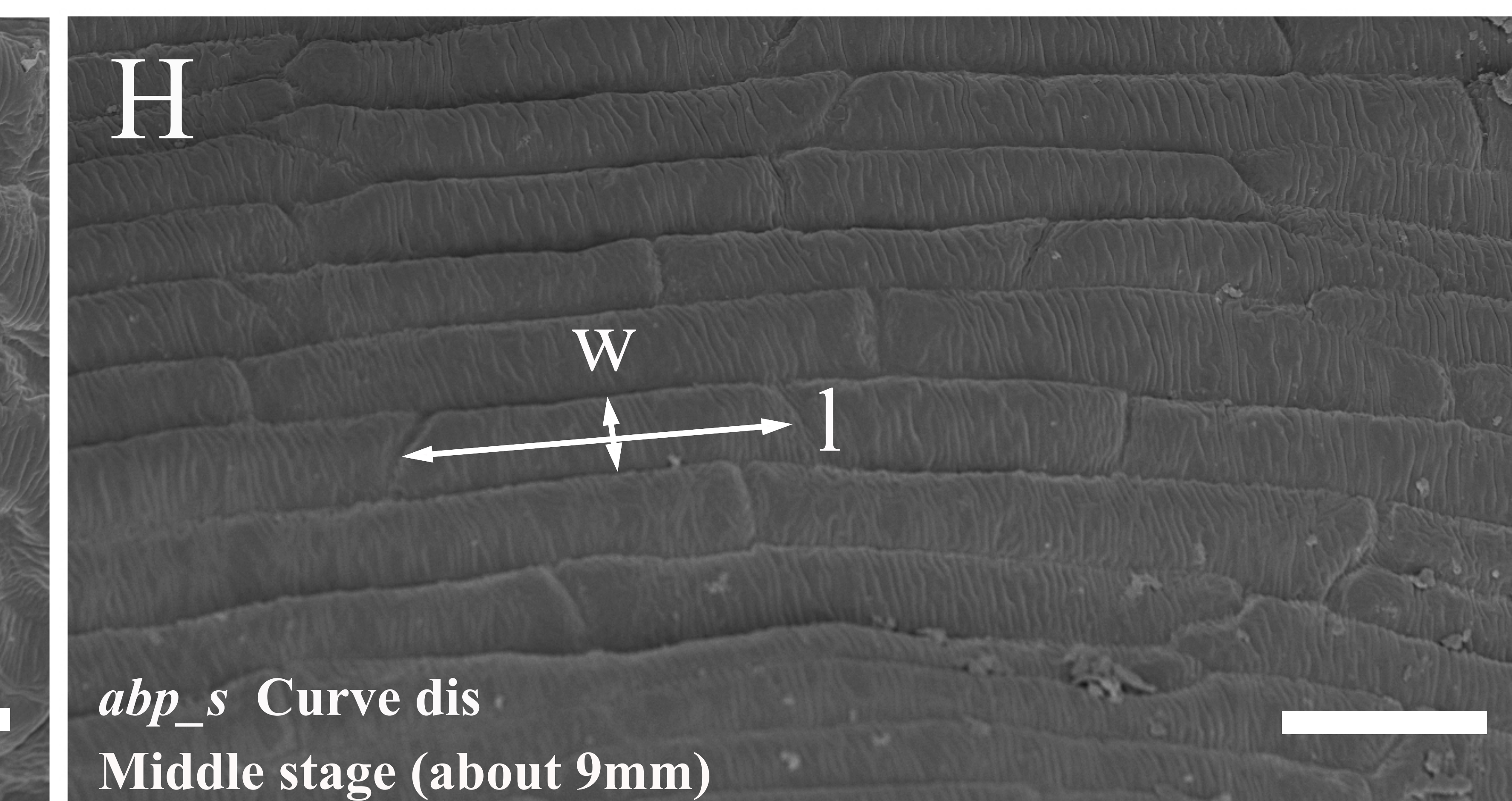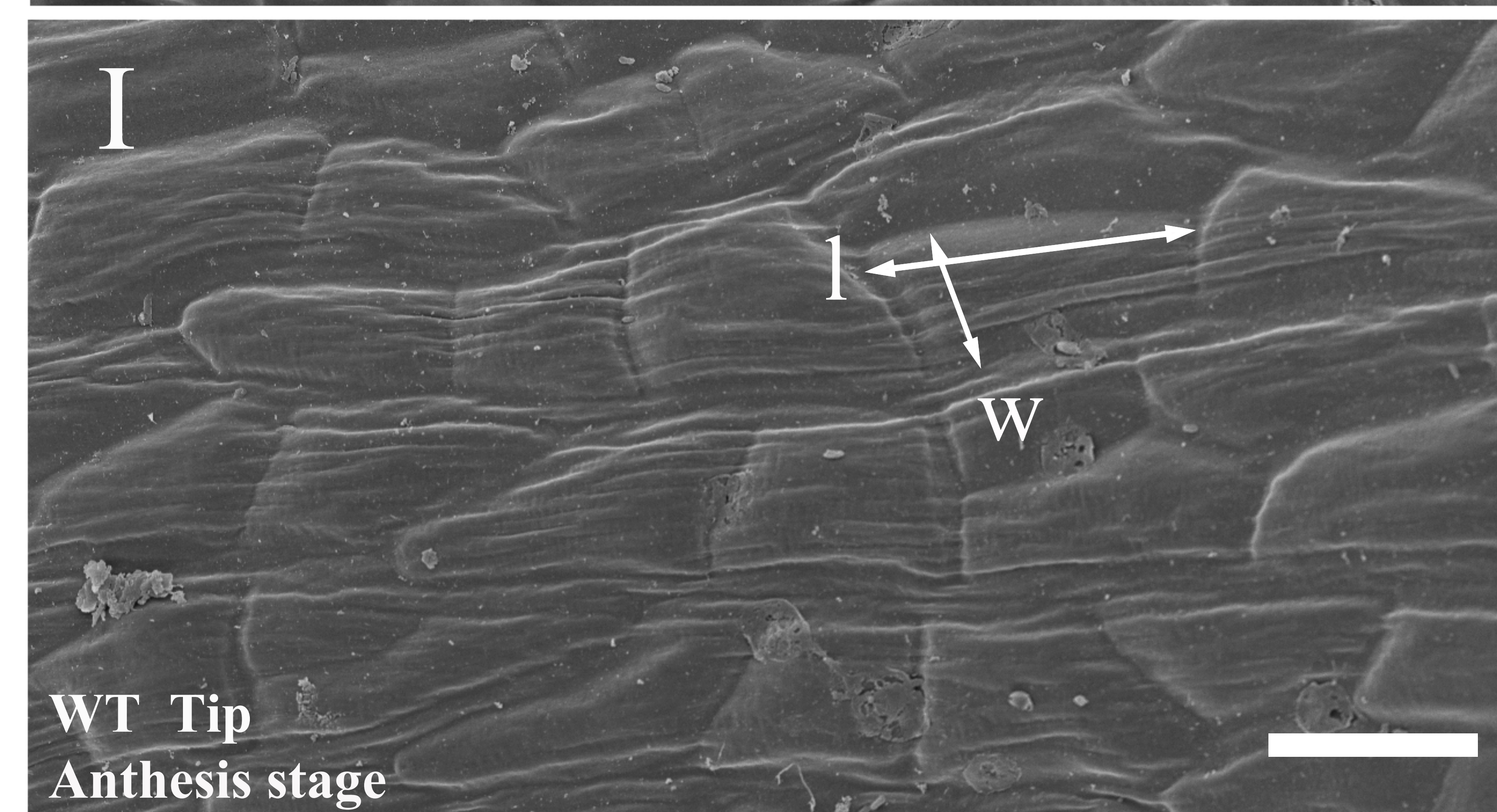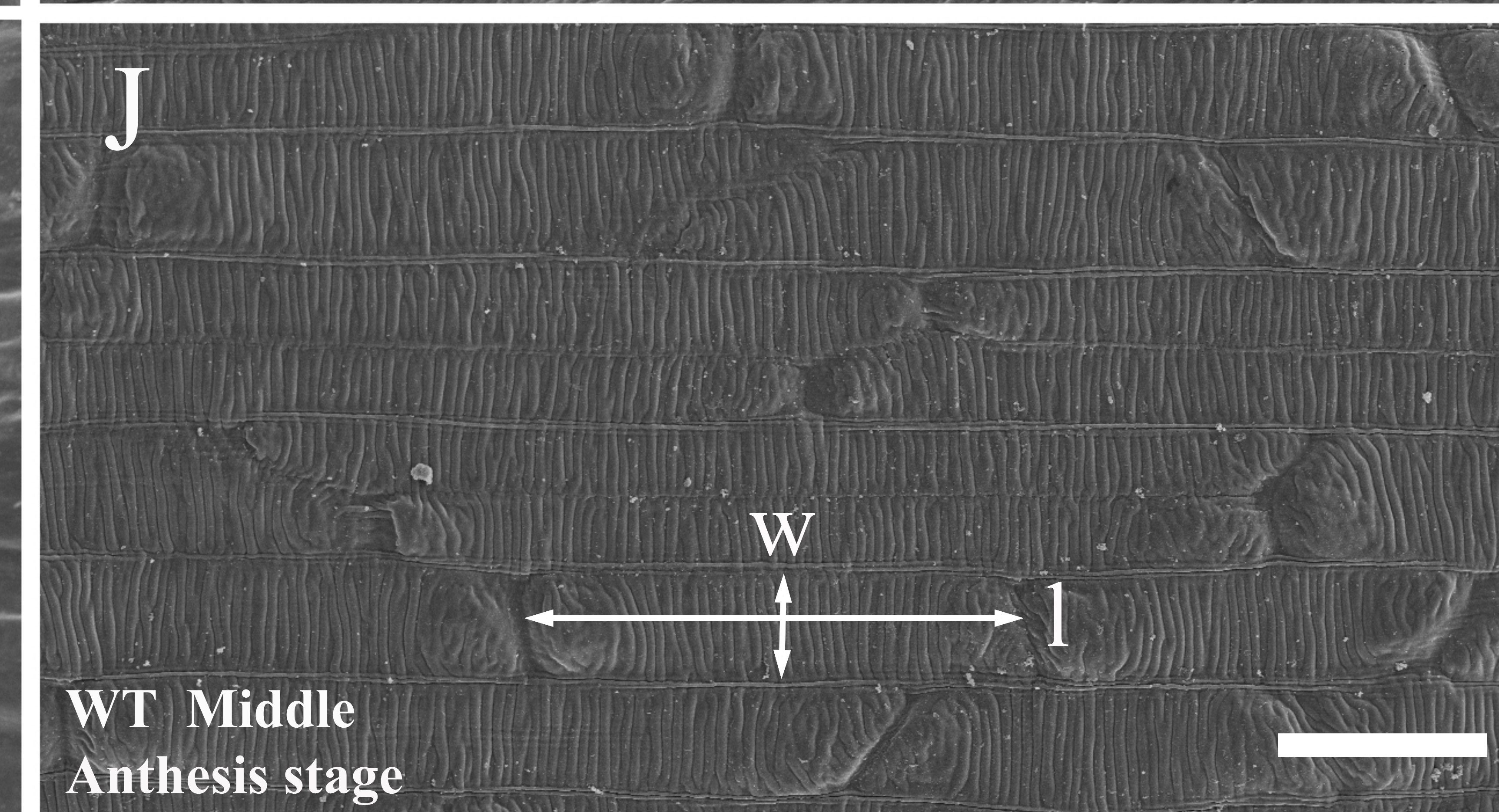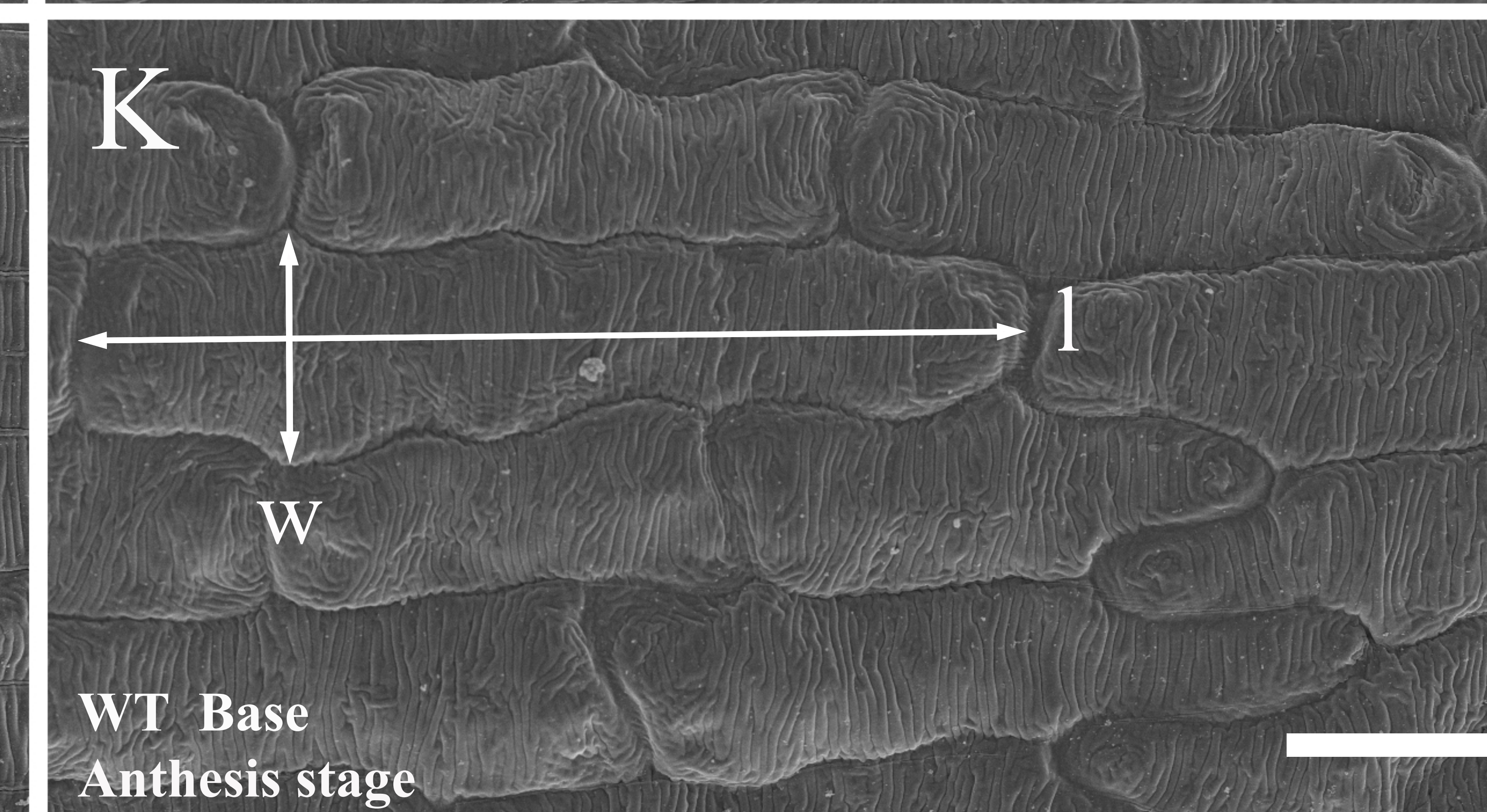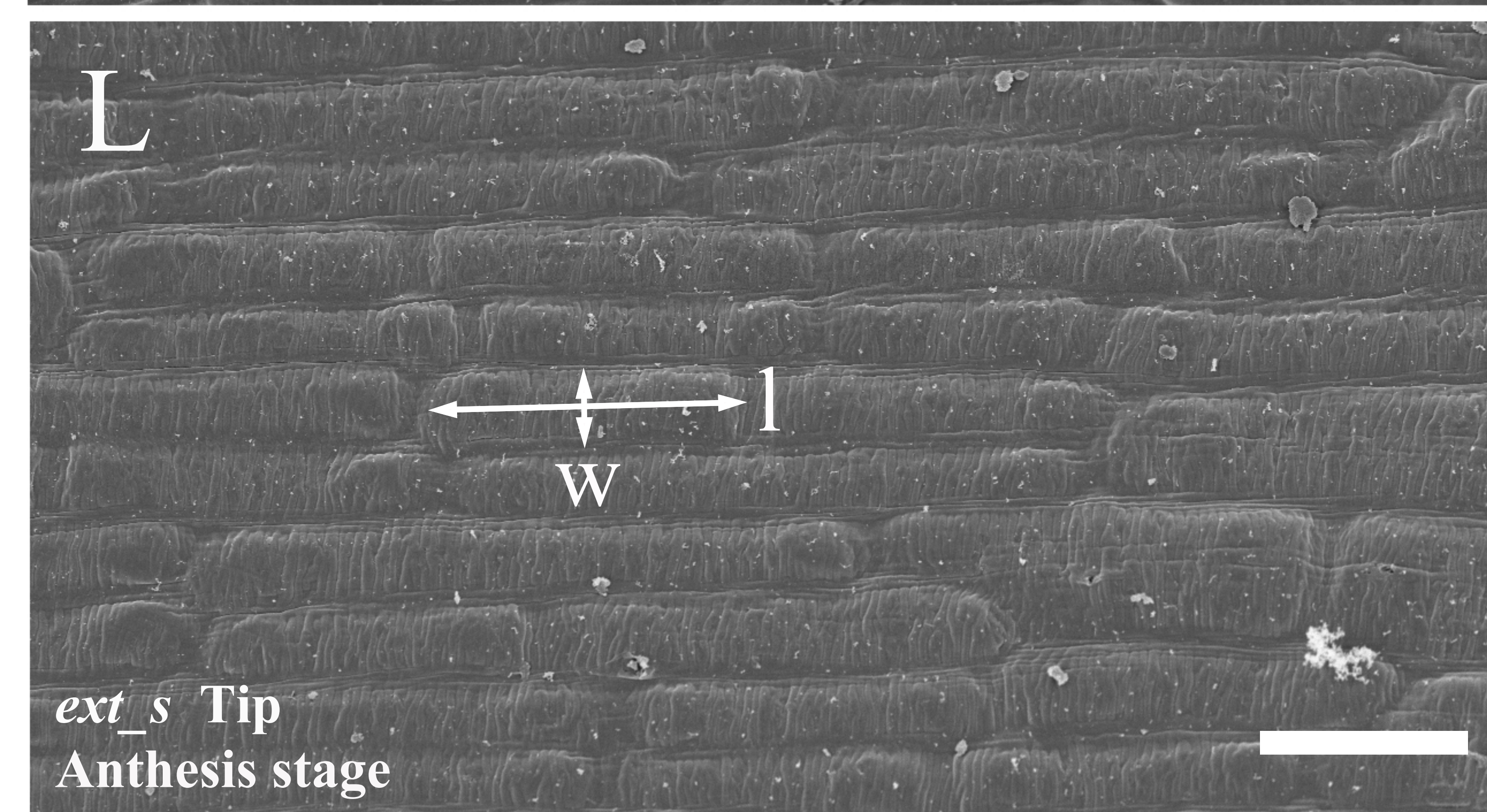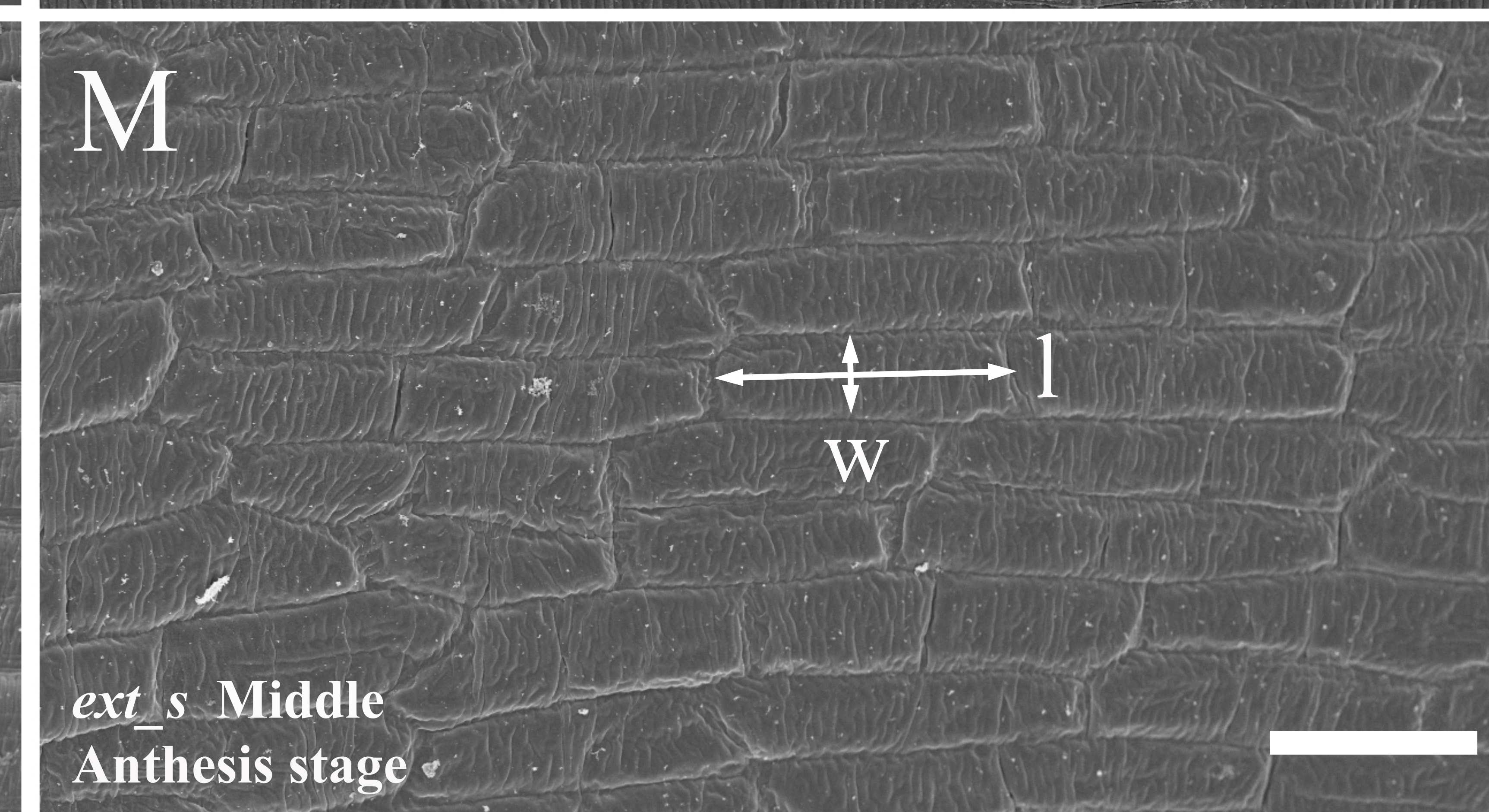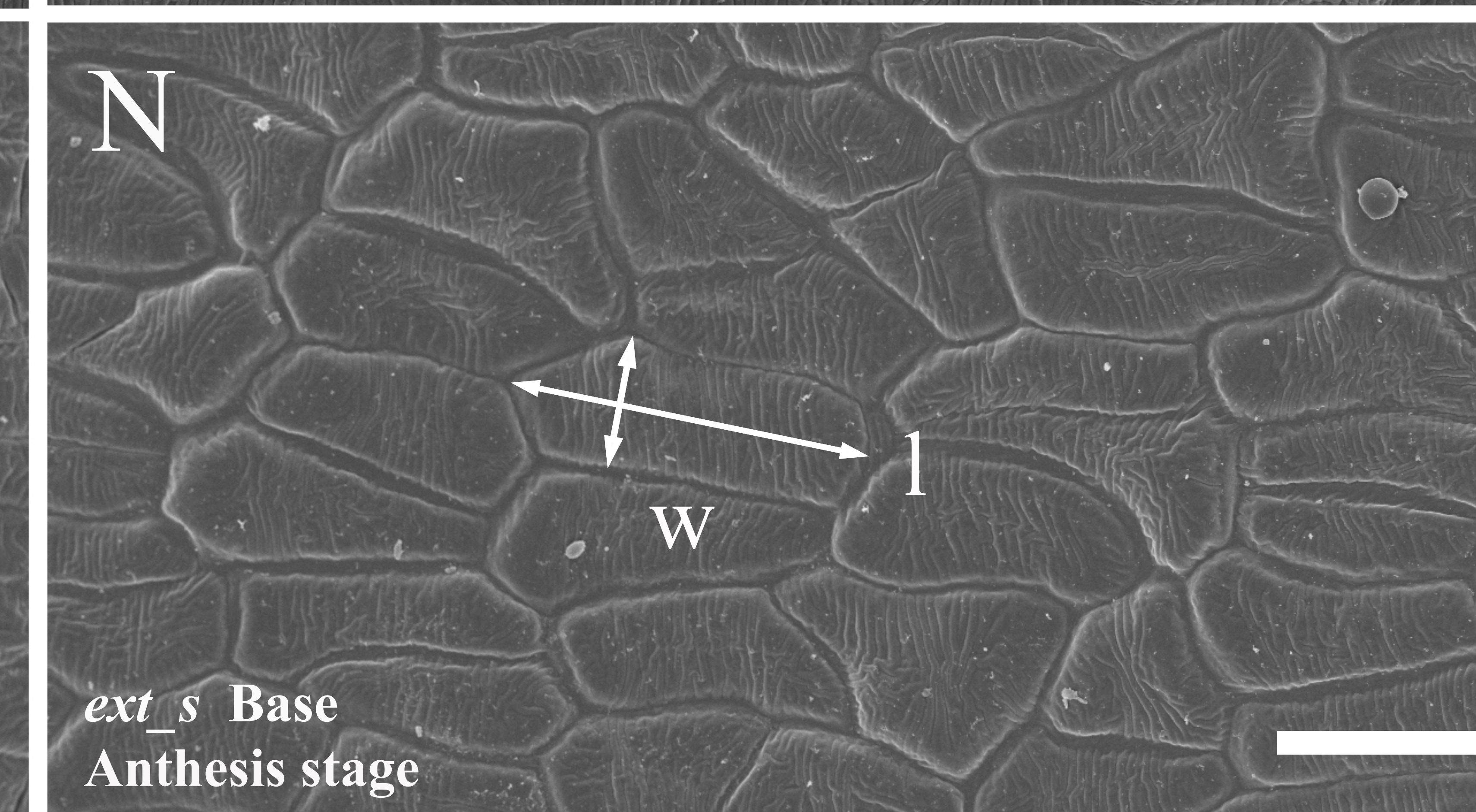

Supplement: Web_Material_uhae015 [file web_material_uhae015.zip › fig S4.pdf]

A

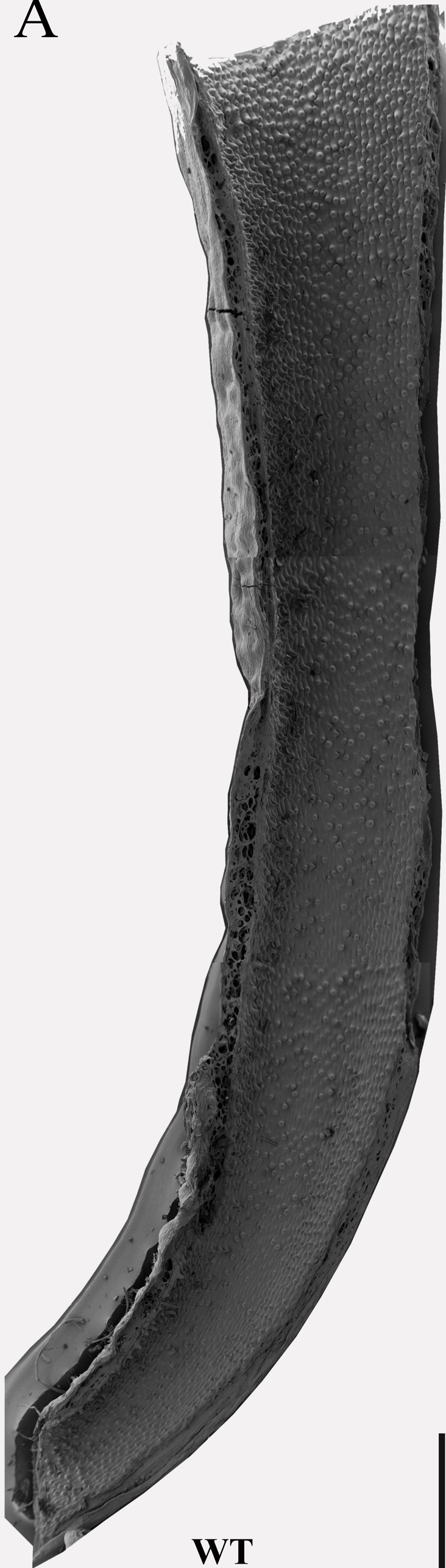

WT

B

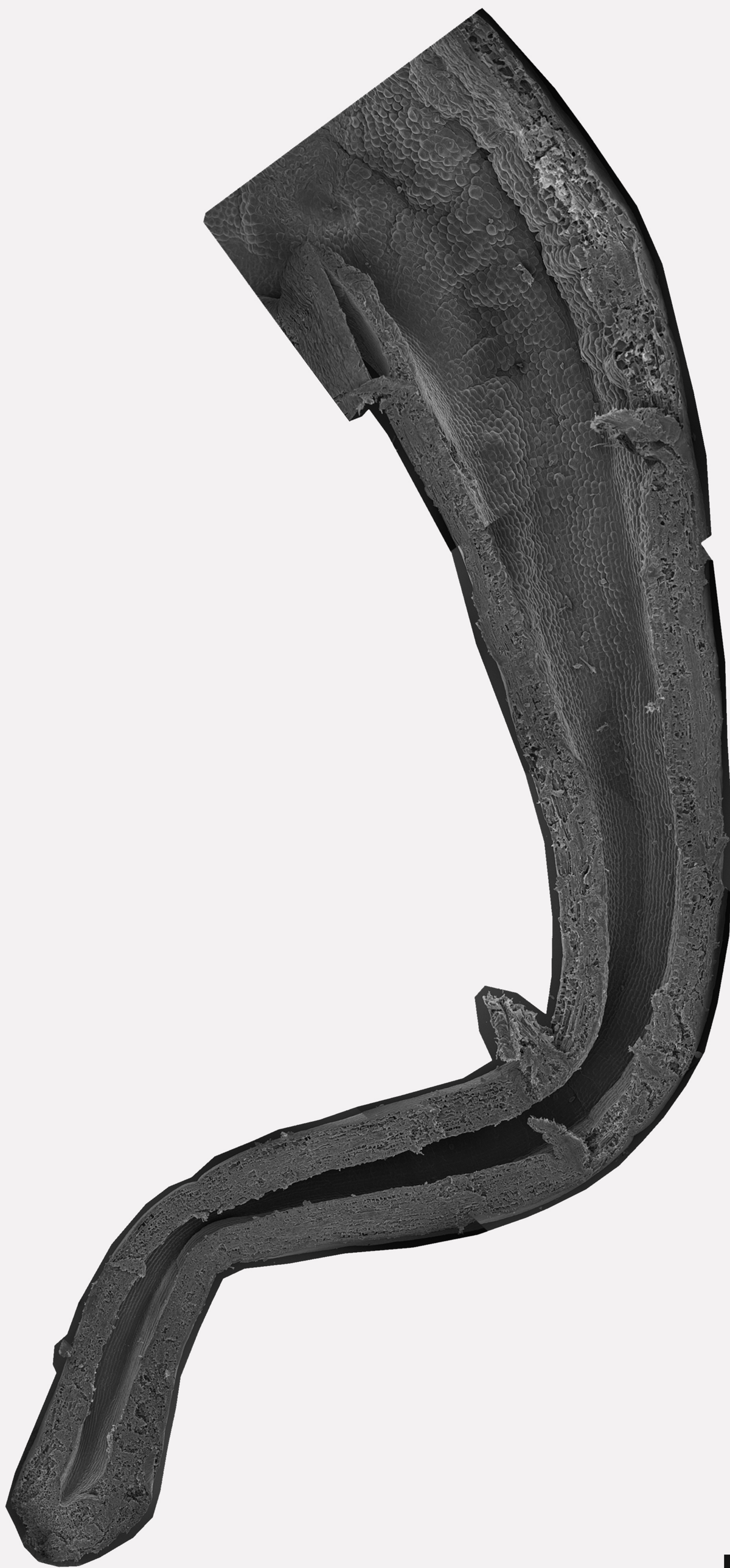

*ext\_s*

C

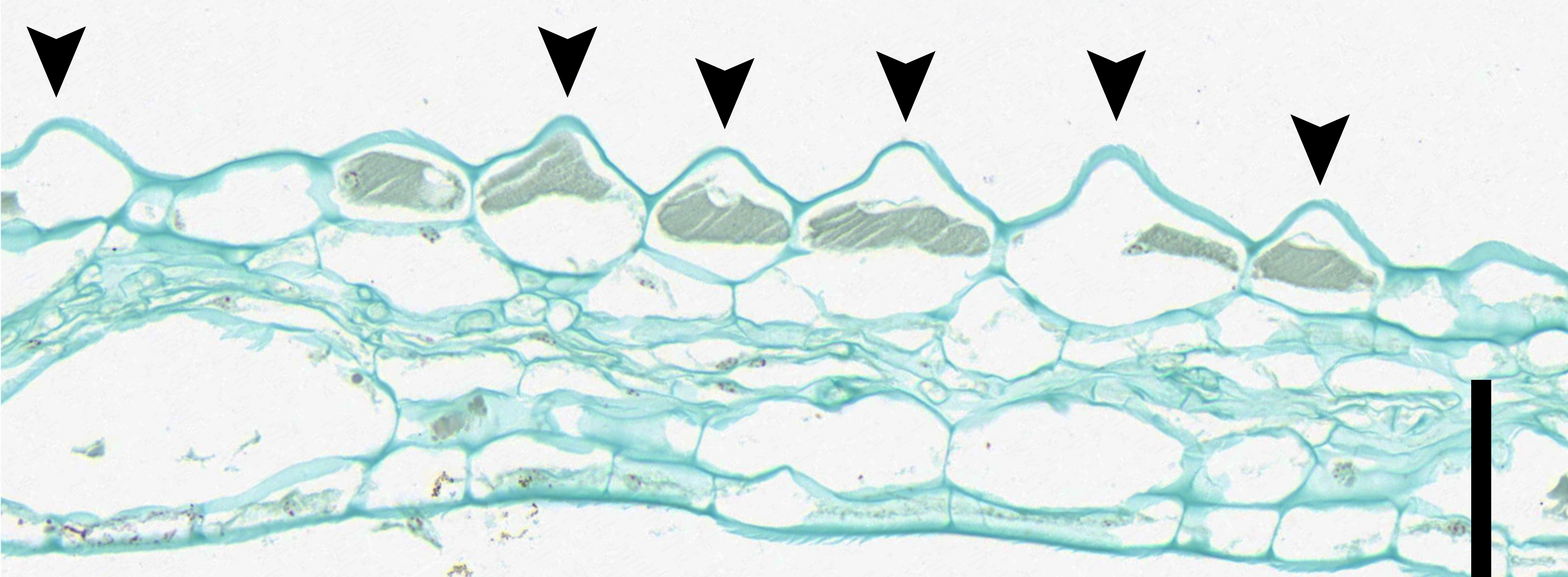

WT

D

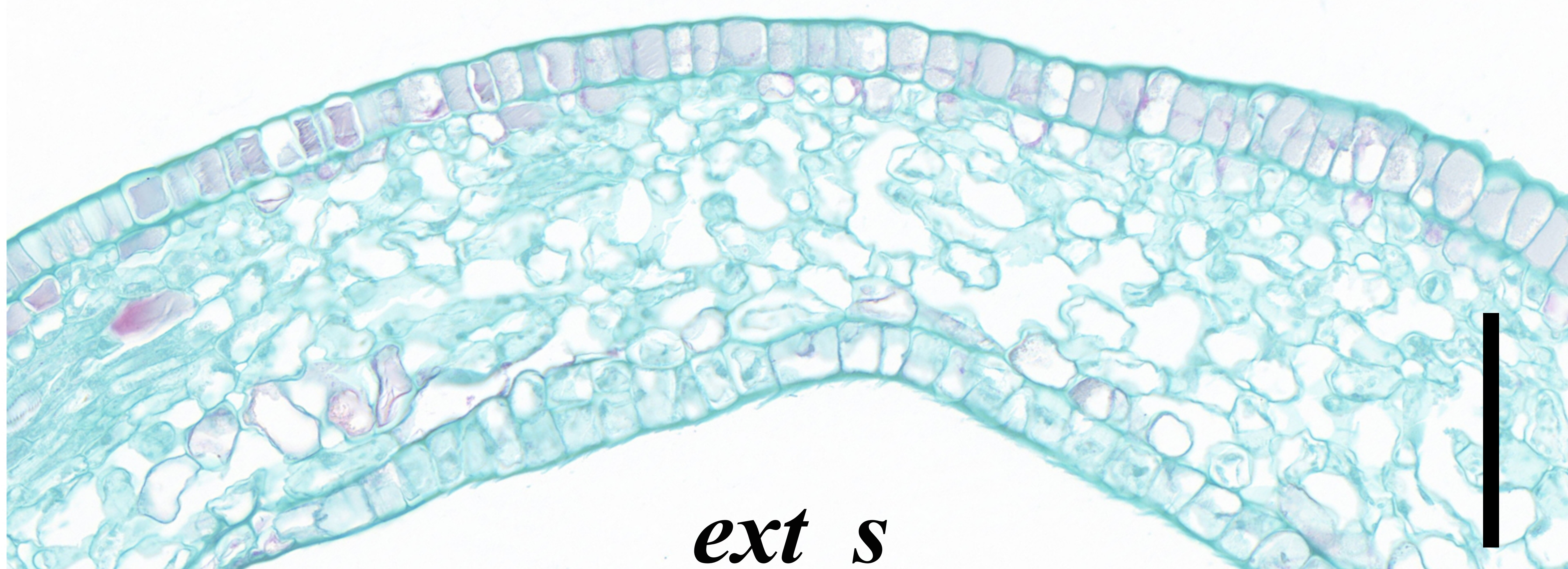

*ext\_s*

Supplement: Web_Material_uhae015 [file web_material_uhae015.zip › Fig S5.pdf]

**A**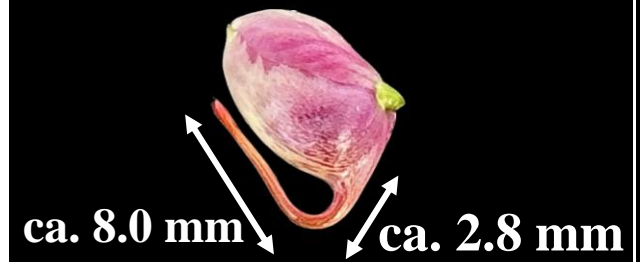**B**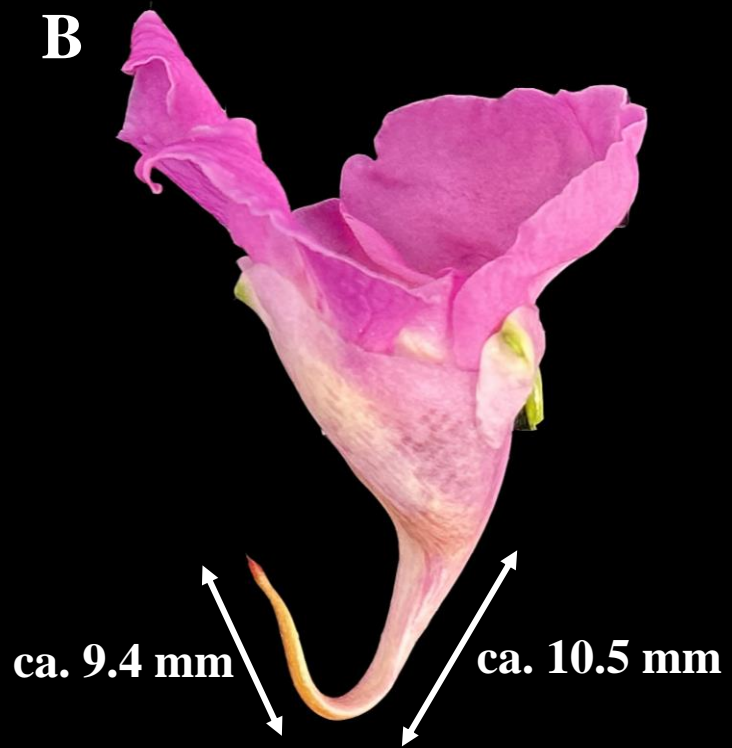**C**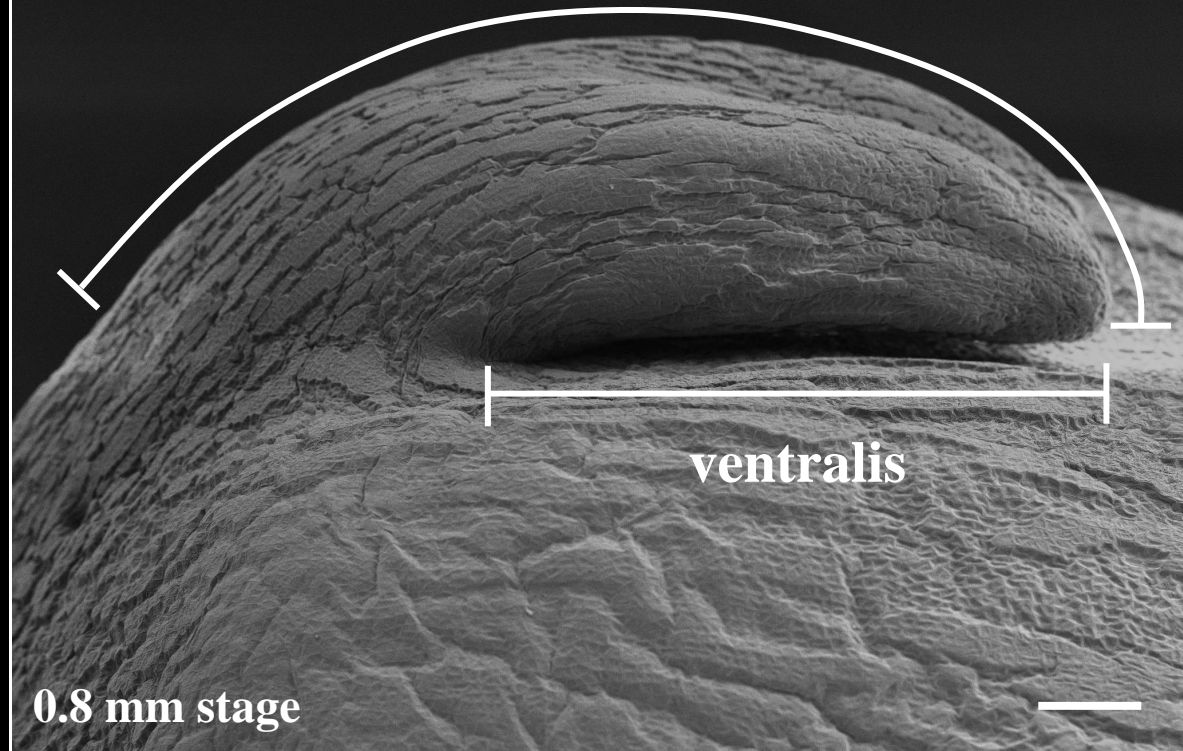

Supplement: Web_Material_uhae015 [file web_material_uhae015.zip › fig S6.pdf]

**A**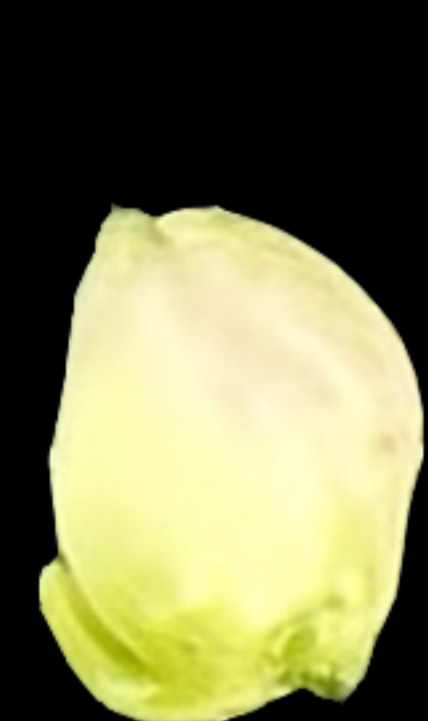**stage 1**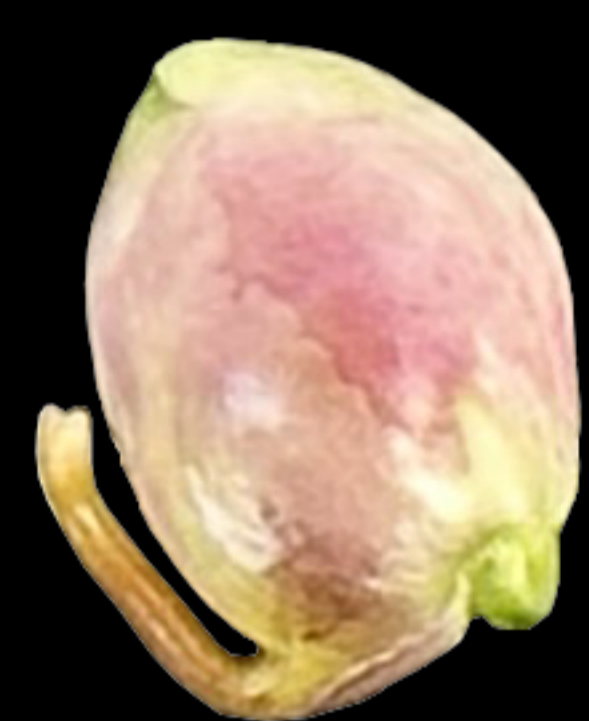**stage 2**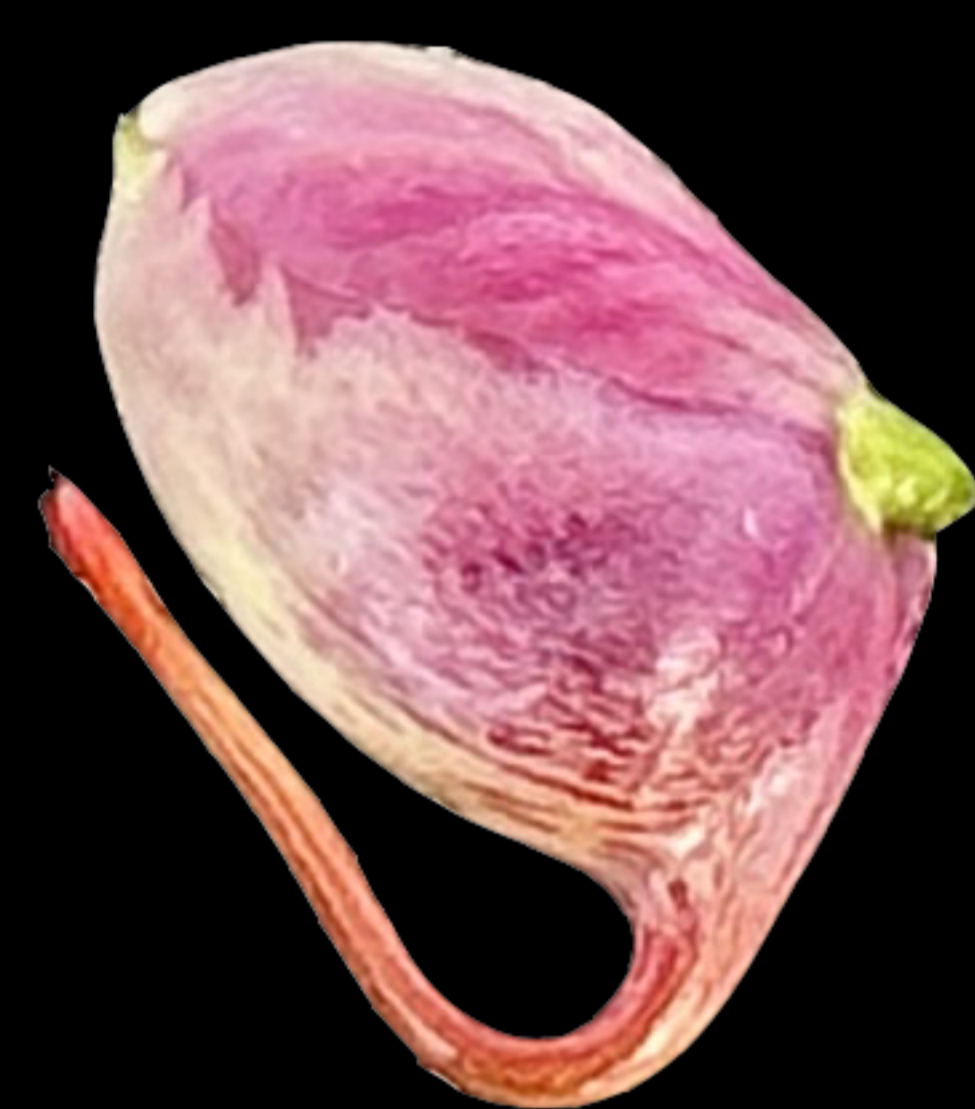**stage 3**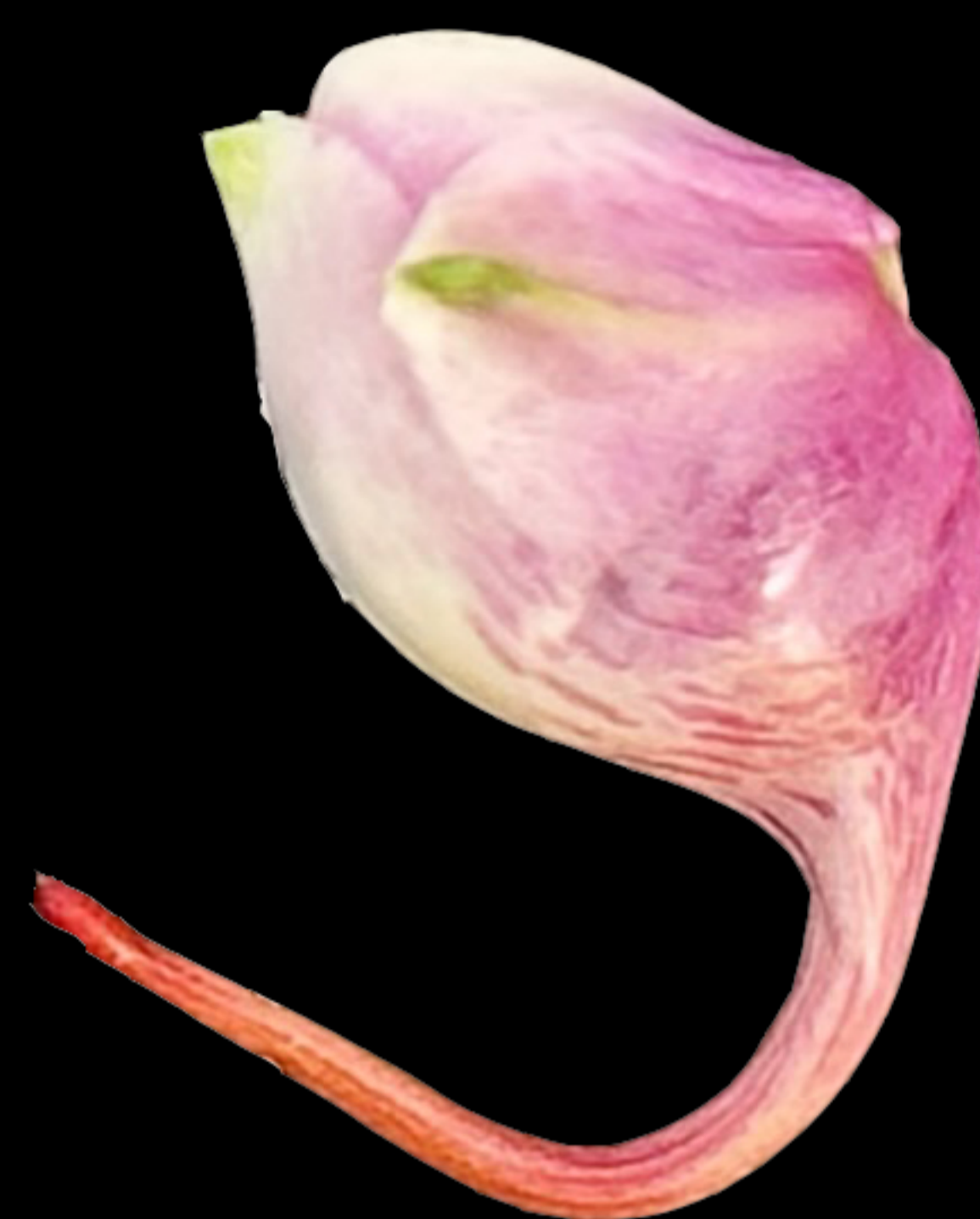**stage 4**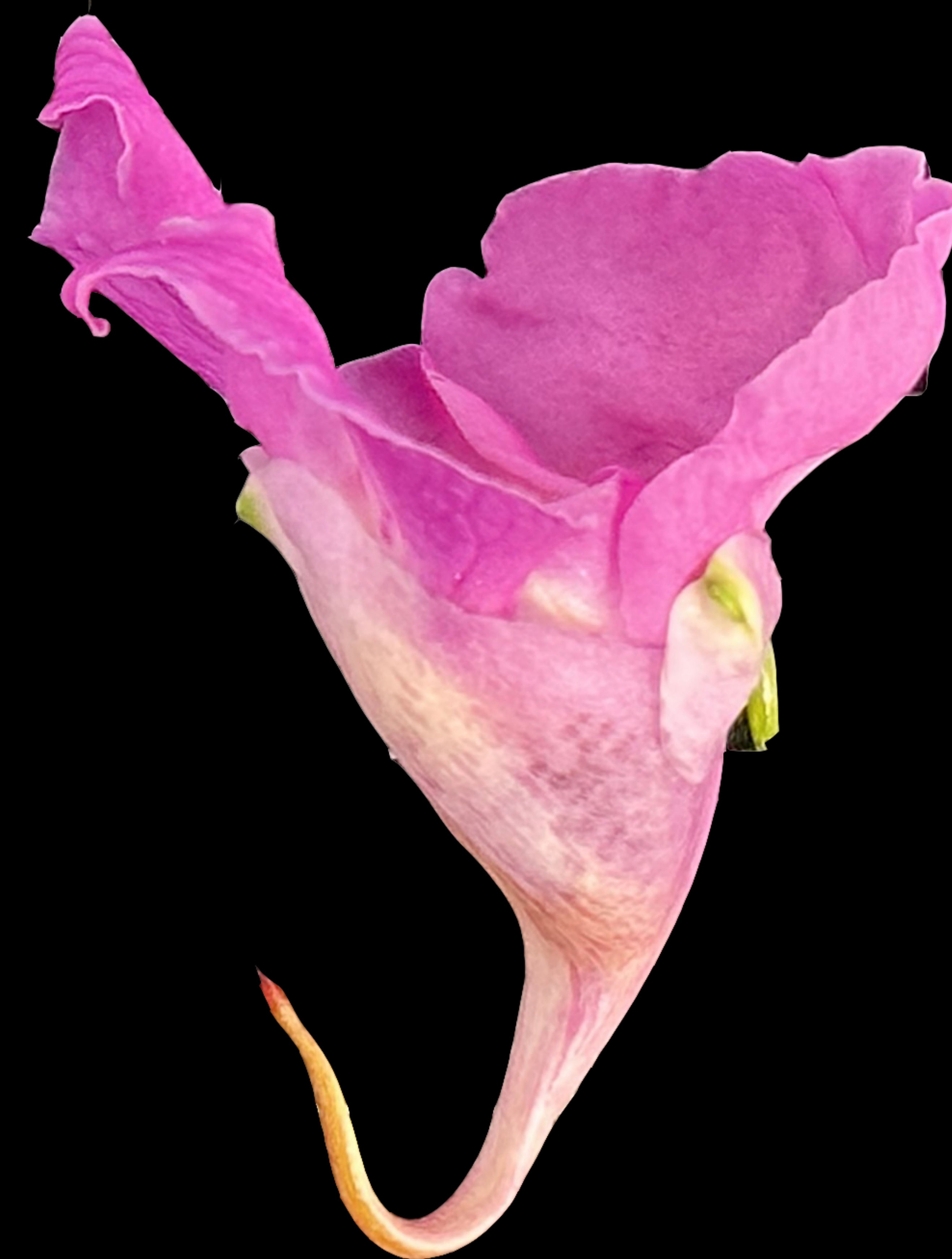**stage 5**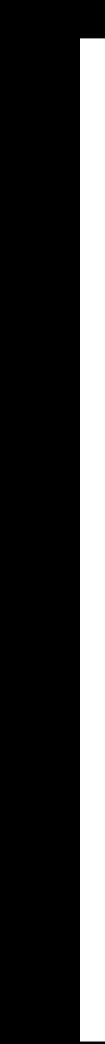**B**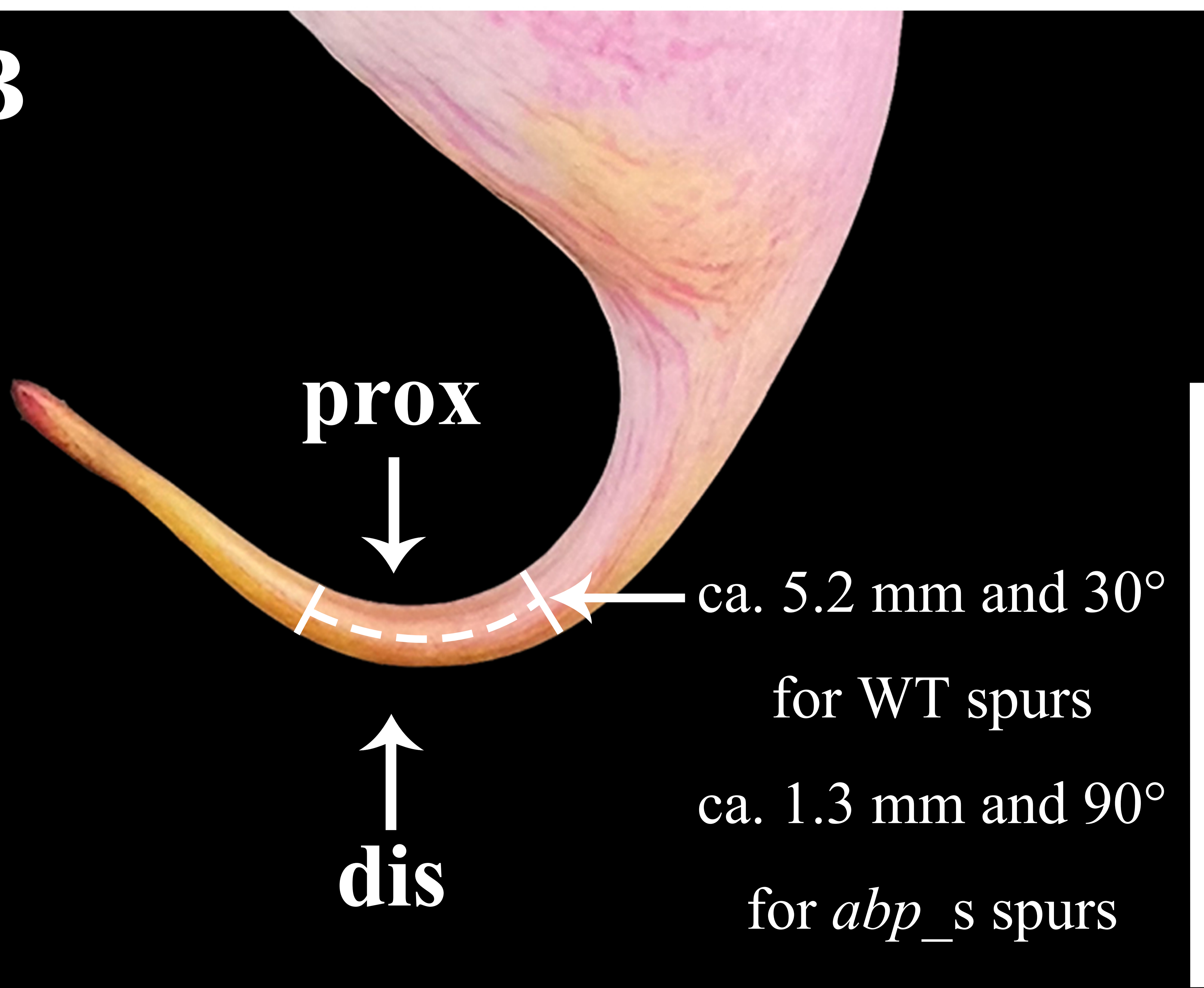**C**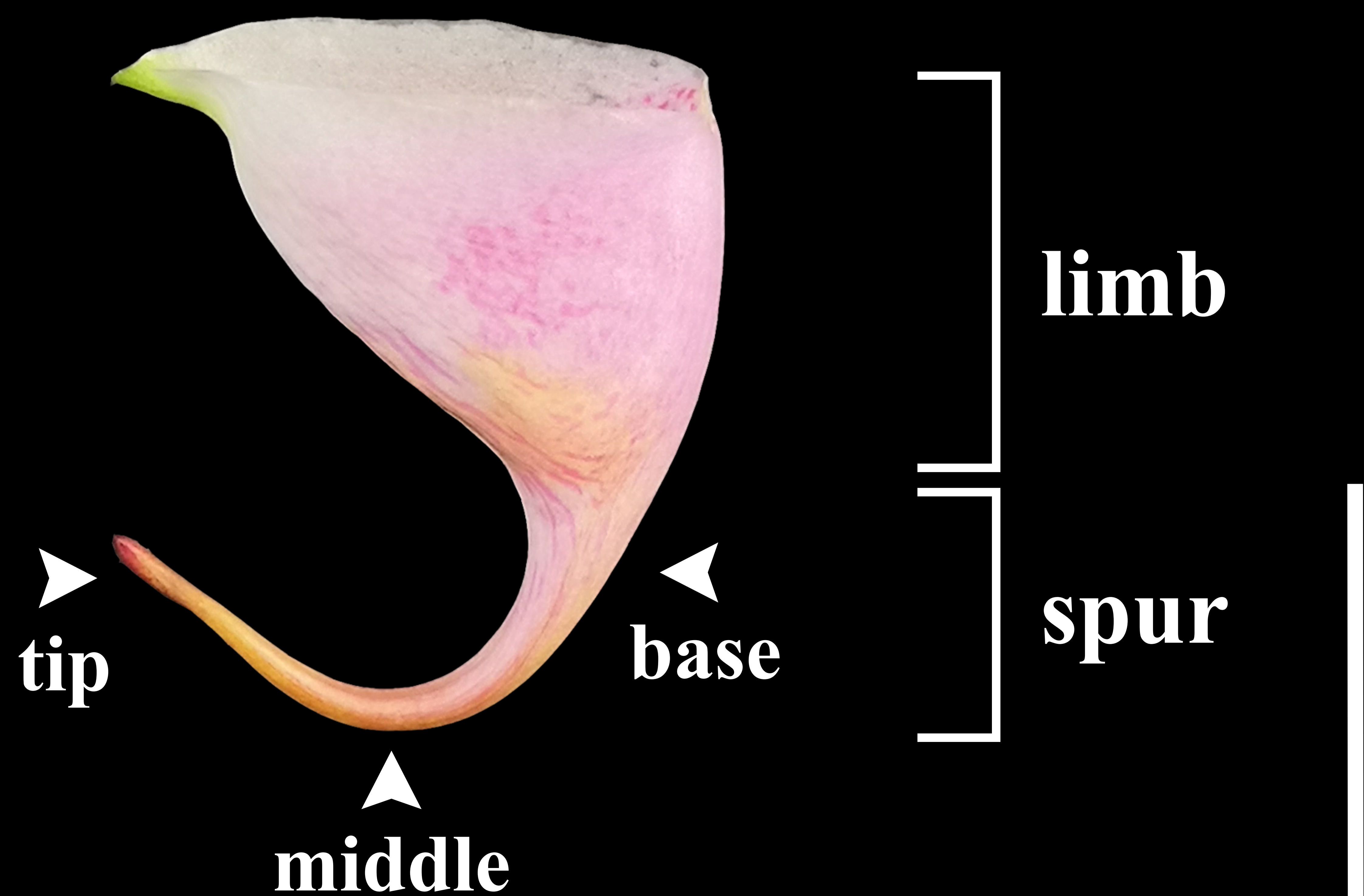

Supplement: Web_Material_uhae015 [file web_material_uhae015.zip › fig S7.pdf]
